# Synthesis of graded CdS_1−*x*_Se_*x*_ nanoplatelet alloys and heterostructures from pairs of chalcogenoureas with tailored conversion reactivity†

**DOI:** 10.1039/d3sc03384h

**Published:** 2023-10-16

**Authors:** Natalie Saenz, Leslie S. Hamachi, Anna Wolock, Berit H. Goodge, Alexis Kuntzmann, Benoit Dubertret, Isabel Billinge, Lena F. Kourkoutis, David A. Muller, Andrew C. Crowther, Jonathan S. Owen

**Affiliations:** a Department of Chemistry, Columbia University New York NY USA jso2115@columbia.edu; b Department of Chemistry, Barnard College, Columbia University New York NY USA; c School of Applied and Engineering Physics, Cornell University Ithaca NY 14853 USA; d Kavli Institute at Cornell for Nanoscale Science, Cornell University Ithaca NY 14853 USA; e Ecole Supérieure de Physique et de Chimie Industrielle Paris France

## Abstract

A mixture of *N*,*N*,*N*′-trisubstituted thiourea and cyclic *N*,*N*,*N*′,*N*′-tetrasubstituted selenourea precursors were used to synthesize three monolayer thick CdS_1−*x*_Se_*x*_ nanoplatelets in a single synthetic step. The microstructure of the nanoplatelets could be tuned from homogeneous alloys, to graded alloys to core/crown heterostructures depending on the relative conversion reactivity of the sulfur and selenium precursors. UV-visible absorption and photoluminescence spectroscopy and scanning transmission electron microscopy electron energy loss spectroscopy (STEM-EELS) images demonstrate that the elemental distribution is governed by the relative precursor conversion kinetics. Slow conversion kinetics produced nanoplatelets with larger lateral dimensions, behavior that is characteristic of precursor conversion limited growth kinetics. Across a 10-fold range of reactivity, CdS nanoplatelets have 4× smaller lateral dimensions than CdSe nanoplatelets grown under identical conversion kinetics. The difference in size is consistent with a rate of CdSe growth that is 4× greater than the rate of CdS. The influence of the relative sulfide and selenide growth rates, the duration of the nucleation phase, and the solute composition on the nanoplatelet microstructure are discussed.

## Abstract 2

A mixture of *N*,*N*,*N*′-trisubstituted thiourea and cyclic *N*,*N*,*N*′,*N*′-tetrasubstituted selenourea precursors were used to synthesize three monolayer thick CdS_1−*x*_Se_*x*_ nanoplatelets in a single synthetic step.

## Graphical abstract

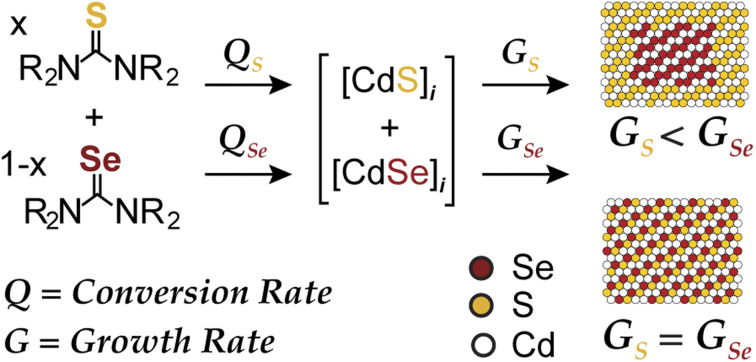

## Introduction

Colloidal nanoplatelets (NPLs) of II–VI semiconductors boast narrow emission linewidths due to one dimensional quantum confinement and atomically precise thickness.^1^ Their giant harmonic oscillator strength, low Auger recombination kinetics, low exciton-phonon coupling, and plane polarized emission make NPLs well-suited to luminescent display, laser, and solid state lighting applications.^2^ The photoluminescence quantum yields (PLQY) of such structures can be increased by growing CdSe/CdS core/crown and CdSe/CdS core/shell heterostructures.^3,4^ These architectures shield photoexcited charges from surface states and soften the exciton confinement potential,^5^ reducing their Auger recombination kinetics.^6^ Grading the composition at the S/Se interface could further reduce Auger recombination and lead to even higher levels of performance in high flux applications.^3,7^ Controlling the alloy composition also provides access to luminescence wavelengths between those of the pure phase platelet thicknesses.^8^ For these reasons, precisely tailored alloys with graded compositions could reach new levels of luminescence performance.^9,10^

**Scheme 1:**
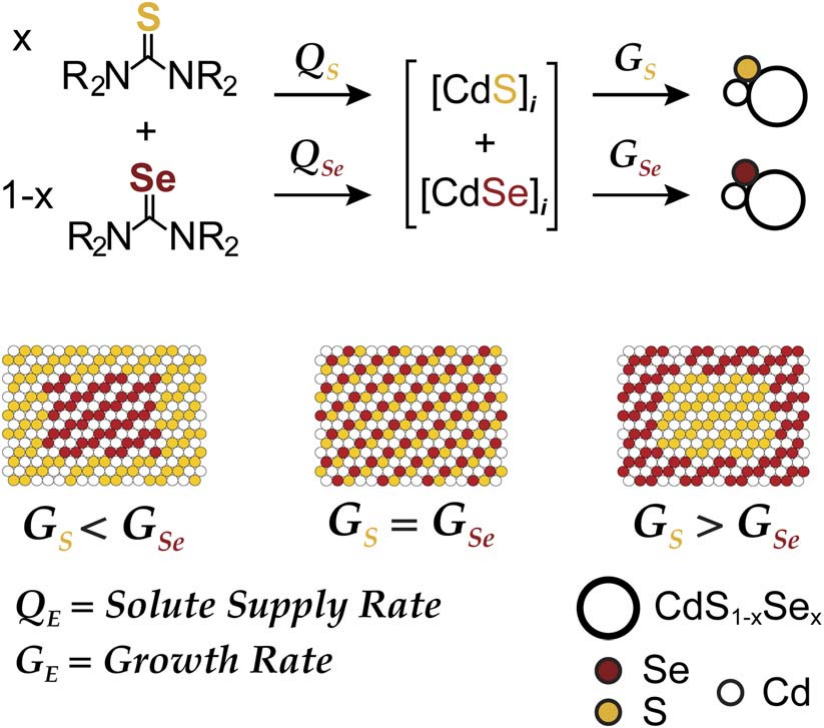
Solute supply kinetics (*Q*) and growth kinetics (*G*) on the microstructure of CdS_1−*x*_Se_*x*_ nanocrystal alloys prepared from a pair of chalcogenoureas.

We recently reported a synthesis of quasi-spherical alloyed nanocrystals (CdS_1−*x*_Se_*x*_) whose microstructure could be tuned using pairs of sulfide and selenide precursors with controlled conversion kinetics (Scheme 1). By adjusting the relative precursor reactivity (*Q*_s_*vs. Q*_Se_), the solute composition during the growth can be controlled and used to influence the relative growth rates (*G*_s_*vs. G*_Se_) and the final microstructure. Core–shell and alloyed nanocrystals were synthesized across a range of sizes and wavelengths and used to produce spherical quantum wells for solid state lighting applications.^10,11^ In the present manuscript we explore a similar approach to synthesize laterally graded nanoplatelets.

To tailor the composition of nanoplatelets using the framework depicted in Scheme 1 two conditions must be met: (1) the crystallization kinetics must be limited by the precursor conversion kinetics and (2) the nucleation process must occur in a burst and prior to any changes in the evolving solute composition. While previous descriptions of nanoplatelet formation indicate that growth proceeds by lateral extension of a nanoplatelet nucleus,^12,13^ the supply of monomers is typically thought to arise from Ostwald ripening,^13,14^ rather than rate limiting conversion of precursor reagents.^15^ Hence, we sought novel precursor reactivity to access a precursor conversion limited growth regime.

Although it is widely accepted that a burst of nucleation is required to obtain narrow size distributions, recent work on the nucleation of InP,^16^ PbS and PbSe,^17,18^ CdSe,^19^ and Ir^20^ provide clear examples where this assumption is incorrect. Instead nucleation kinetics can be slow and occur over a significant fraction of the total reaction without compromising the monodispersity.^16,18^ However, if the ratio of S : Se solutes evolves during the nucleation phase, those crystals nucleated at early times can have different S : Se compositions than those nucleated at later times. Such behavior could explain the broad polydispersity of quasi-spherical CdS_1−*x*_Se_*x*_ nanocrystals prepared from pairs of thio- and selenoureas whose conversion reactivity differs less than 10×.^11^ On the other hand, a short burst of nucleation at the beginning of the synthesis could allow the composition of the entire ensemble of crystals to evolve with a changing solute composition.

Nanoplatelets appear to nucleate in a shorter period than spherical quantum dots. This hypothesis is consistent with the low polydispersity of typical nanoplatelet edge lengths, (which we reason is less likely to result from size dependent growth kinetics) and their large dimensions (nanoplatelets studied herein are composed of 5000–100 000 CdE units *versus* 200–1000 CdE units for spherical quantum dots). Nanoplatelet nucleation may, therefore, be better suited to the mixed precursor approach described in Scheme 1.

Nanoplatelet optical properties provide a useful tool to investigate the relationship between the solute composition and the microstructure. When monitoring the growth of quasi spherical CdS_1−*x*_Se_*x*_ quantum dots from mixtures of sulfur and selenium precursors, quantum confinement and the alloy composition both influence the absorption and luminescence spectrum. The conflation of these effects makes it challenging to study the correlation between the solute supply kinetics and alloy microstructure. The optical absorption spectrum of CdS_1−*x*_Se_*x*_ nanoplatelets, on the other hand, is insensitive to changes in the lateral dimensions, producing spectra that are primarily determined by the alloy composition. As a result, the alloy microstructure is more readily ascertained from the optical spectrum.

The elemental composition of nanoplatelets has previously been graded in the thickness direction by the slow injection of tri-*n*-octylphosphine-chalcogenides^21^ and layer-by-layer methods such as colloidal atomic layer deposition (c-ALD).^22,23^ Only one report describes the formation of laterally graded alloys from elemental sulfur and selenium dissolved in octadecene.^9^ However, it is unclear how the relative reactivity of those chalcogens with octadecene influence the solute composition over time and the difficulty of characterizing the spatial distribution of elements makes it challenging to relate the optical properties to the nanoplatelet microstructure.

Here we use precisely controlled precursor conversion kinetics to infer the steady state solute concentration and predict the nanoplatelet composition. The framework illustrated in Scheme 1 captures the evolution of the nanoplatelet composition with time, allowing the spectral properties and nanoplatelet composition to be correlated.

## Results and discussion

### Model synthesis

**Fig. 1:**
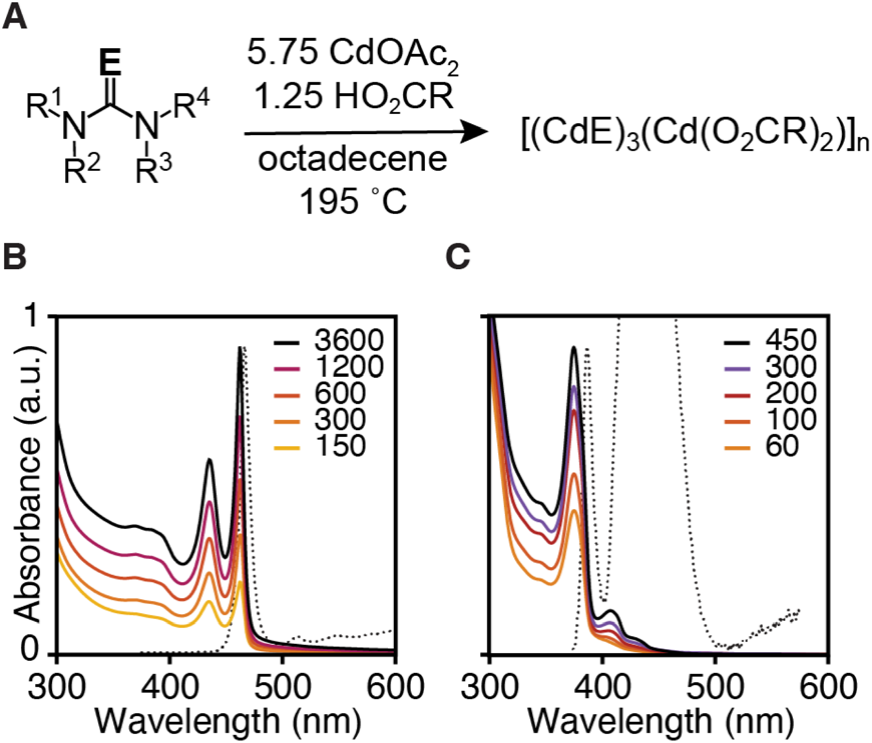
(A) Synthesis conditions used to prepare 3 ML nanoplatelets of CdSe (B) and CdS (C). Legend displays time with units of seconds at which quantitative aliquots were taken.

Three monolayer thick CdS and CdSe nanoplatelets (3 ML) were synthesized using *N*,*N*,*N*′-trisubstituted thioureas and cyclic tetrasubstituted selenoureas, cadmium acetate dihydrate, and oleic acid in 1-octadecene (ODE) at 195 °C (Fig. 1 and Table 1). By selecting the appropriate precursor, the kinetics of solute supply can be designed to limit the crystal nucleation and growth kinetics.^11,24,25^ Under these conditions, nanoplatelets are formed without significant contamination by other nanocrystals, which are well known to limit the nanoplatelet yield under traditional conditions.^26,27^

**Table 1:** Chalcogen precursors used in this study and their conversion reaction coefficients (*k*_obs_) under conditions shown in Fig. 1

	E	R^1^	R^2^	R^3^	R^4^	*k*_obs_ (s^−1^)
1	S	H	*n*-C_6_H_13_	H	*n*-C_12_H_25_	a
2	S	H	*n*-C_6_H_13_	*n*-C_8_H_17_	*n*-C_8_H_17_	a
3	S	H	Cy	–(CH_2_)_4_–		a
4	S	H	*n*-C_6_H_13_	–(CH_2_)_4_–		a
5	S	H	Ph	–(CH_2_)_4_–		0.0329
6	S	H	Bn	–(CH_2_)_4_–		0.0179
7	S	H	Ph	*n*-C_4_H_9_	*n*-C_4_H_9_	0.0176
8	S	H	Ph	CH_3_	Ph	0.00668
9	S	H	3,5-Me_2_Ph	CH_3_	Ph	0.0145
10	S	H	4-MePh	CH_3_	Ph	0.0129
11	S	H	–CH_2_CH_2_–		H	0.00525
12	S	H	–CH_2_CH_2_–		Ph	0.00121
13	S	H	–CH_2_CH_2_–		Me	0.00167
14	S	H	–CH_2_CH_2_–		Et	0.00124
15	Se	H	–CH_2_CH_2_–		Ph	a
16	Se	H	–CH_2_CH_2_–		Et	0.0134
17	Se	H	–CH_2_CH_2_–		iPr	0.0101
18	Se	Ph	–CH_2_CH_2_–		Ph	0.0149
19	Se	Me	–CH_2_CH_2_–		Me	0.00169
20	Se	Et	–CH_2_CH_2_–		Et	0.00047

a Conversion kinetics too rapid to measure.

The precipitation kinetics are measured using UV-vis absorption spectroscopy and an extinction coefficient (*λ* = 350 nm) that is known to be independent of the nanocrystals size for CdSe or the band edge absorption for CdS.^22,23,28^ By assuming the rate of semiconductor formation is equal to the rate of precursor conversion, a single exponential fit to the absorbance data is used to extract a conversion reactivity coefficient (*k*_obs_) analogous to an observed rate constant (Fig. S1, S2† and Table 1). However, the evolution of the absorbance appears more complex than single exponential in some cases (see Fig. S1 and S2†). Nonetheless, the *k*_obs_ provides a convenient method of distinguishing the relative reactivity of the precursors over the ∼1000-fold range of coefficients measured here. The large range of growth kinetics indicates that the chalcogenourea conversion reactivity limits the formation of the nanoplatelets, as was also observed in a recent study on quasi-spherical nanocrystals.^29^ The reactivity trends observed here are similar to those reported by us earlier and are consistent with a Lewis acid activation mechanism that leads to C=E bond cleavage.^25,26^

Precursors that provide controlled conversion reactivity produce single populations of 3 ML nanoplatelets in good yields. The most reactive selenourea precursors lead to mixtures of 2 ML and 3 ML platelets that slowly evolve into a pure sample of 3 ML platelets. Less reactive selenourea precursors and long reaction times eventually led to small amounts of 4 ML and 5 ML nanoplatelets visible in the photoluminescence emission spectrum of aliquots, presumably *via* a ripening process.

The synthesis of CdS nanoplatelets was less selective for the 3 ML thickness. The most reactive trisubstituted thioureas form mixtures of nanoplatelets with different thicknesses. For example, syntheses beginning from 1 and 2 form 4 ML, and 5 ML CdS nanoplatelets as impurities within 5 minutes of reaction. Less reactive precursors such as the monosubstituted cyclic thiones (11–14) cleanly form 3 ML CdS nanoplatelets. The 3 ML CdS nanoplatelets slowly convert to 4 ML CdS nanoplatelets at much longer reaction times than those used in our kinetics measurements, presumably by a ripening mechanism. The lower selectivity of rapid reactions suggests that the nucleation of the 4th and 5th layers is more rapid at high supersaturation. Nonetheless, in all cases, ripening is much slower than precursor limited growth, and 3 ML nanoplatelets are the major product.

**Fig. 2:**
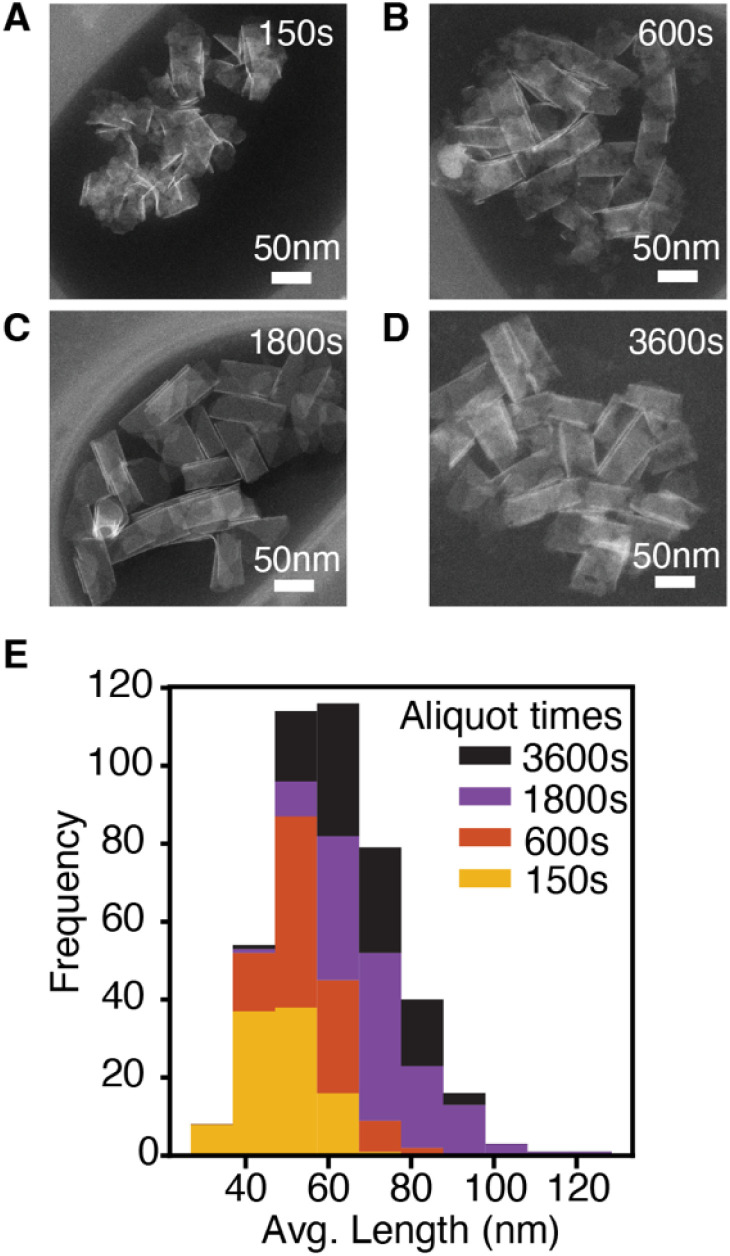
CdSe nanoplatelets from a NPL reaction using 18. (A–D) STEM images of aliquots at reaction time indicated. (E) Histogram of the average nanoplatelet length measured from STEM images.

The lateral dimensions of nanoplatelets were measured throughout the reaction using high angle annular dark field scanning transmission electron microscopy (HAADF-STEM) (Fig. 2, see ESI† for error analysis). These measurements indicate that nanoplatelets grow *via* lateral extension (see ESI†). Example nanoplatelet dimensions observed in aliquots are shown in Fig. 2. The theoretical volume of nanoplatelets measured with HAADF-STEM and the yield measured by UV-vis absorption spectroscopy were used to estimate a nanoplatelet concentration at each time point (Fig. S6†). The nanoplatelet concentrations are stable over the course of the reaction, which is consistent with the formation of a single population of nanoplatelets at early times that is slow to ripen or aggregate. The slow kinetics of ripening/aggregation simplifies our analysis of the spectral evolution of alloyed platelets described below.

### Nucleation and growth kinetics and mechanism

Having established conditions that produce 3 ML nanoplatelets under conditions where the precursor conversion limits the growth, we probed the relationship between the nucleation kinetics and the precursor reactivity. When the rate of solute supply (*Q*_E_) is slower than the rate of solute consumption by crystal nucleation and growth (*G*_E_), the extent of nucleation becomes sensitive to the precursor reactivity (Scheme 1). Such a relationship has never been demonstrated for nanoplatelets, although it is well known in the growth of quasi-spherical and rod shaped quantum dots.^11,16,18,24,25,28–30^ Conventional nanoplatelet syntheses, on the other hand, control the lateral dimensions by changing the temperature, terminating the synthesis at partial yield, or by changing the surfactant structure.^9,26,31^

**Fig. 3:**
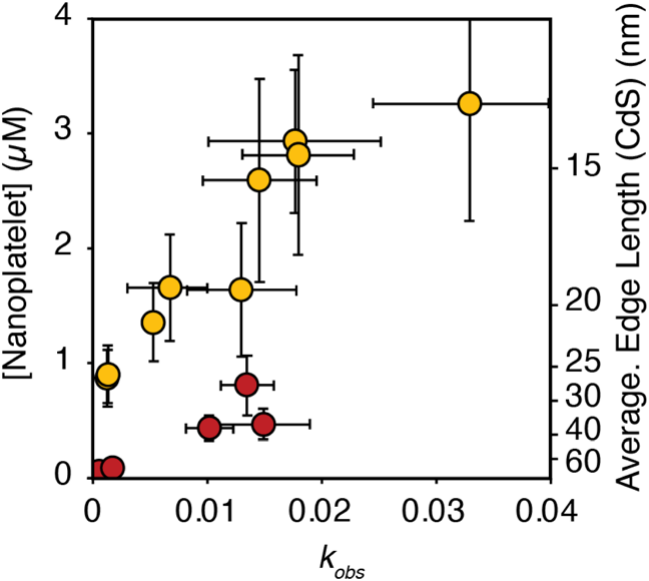
Theoretical nanoplatelet concentration (yellow = CdS, red = CdSe), determined from the nanoplatelet size as measured by TEM and assuming 100% yield of CdE (10 mM) as nanoplatelets, *versus* the associated precursor *k*_obs_. The average edge length of CdS nanoplatelets as measured by TEM is shown on the right axis. Error in the nanoplatelet concentration is propagated from the standard deviation in the average length as measured by TEM (see ESI†). Note, that the right hand axis only applies to the edge length of CdS. CdSe nanoplatelets are 4% larger than the axis indicates.

The influence of *k*_obs_ on the final nanoplatelet dimensions and nanoplatelet concentration is shown in Fig. 3. It can be seen that the concentration of nanoplatelets increases with the conversion reactivity. The relationship is consistent with a homogeneous nucleation and growth mechanism as described using nucleation mass balance models.^32^ Interestingly, at a given value of *k*_obs_, the number of CdS nanoplatelets is ∼4 times greater than the number of CdSe nanoplatelets. Similar behavior is observed when synthesizing CdS and CdSe quasi-spherical nanocrystals in the absence of cadmium acetate.^11^

In the absence of Ostwald ripening or aggregation, the number of nanoplatelets is determined by the relative kinetics of growth and nucleation. These two manifolds compete for solutes. Faster growth kinetics effectively divert solutes from the nucleation manifold leading to fewer and larger nanoplatelets. A 4× difference in the growth kinetics of CdSe and CdS can explain their relative extents of nucleation shown in Fig. 3. The lower amount of CdSe nanoplatelets produced at a given solute supply rate suggests that CdSe nanoplatelets grow more rapidly than CdS nanoplatelets under otherwise identical conditions. A similar observation was made in our study of spherical QDs.^11^

A difference in growth kinetics could impact the solute evolution and microstructure in mixed precursor synthesis (Scheme 1). Under conditions where the solute is composed of both sulfide and selenide a 4× faster rate of CdSe attachment will cause CdSe to concentrate toward the nanoplatelet core. While it has been reported that cadmium sulfide attaches more readily than zinc sulfide,^33^ less is known about the relative rate of sulfide and selenide attachment. Detailed measurements of the nanoplatelet composition could, in principle, be used to address this issue.

### One pot heterostructure synthesis

Nanoplatelet heterostructures are synthesized by the simultaneous injection of a pair of thio- and selenoureas in an analogy to our previous report on spherical CdS_1−*x*_Se_*x*_ nanocrystals.^11^ By adjusting the reactivities and concentrations of each precursor the composition of the solute can be controlled and the nanoplatelet composition adjusted from core/crown to alloyed compositions. The temporal evolution of the solute composition during growth can be estimated from the initial precursor concentration, its *k*_obs_, and by assuming the incorporation of solutes is unselective for sulfide or selenide. This approximation is qualitatively consistent with the evolution of nanoplatelet absorbance and the photoluminescence as described below.

**Fig. 4:**
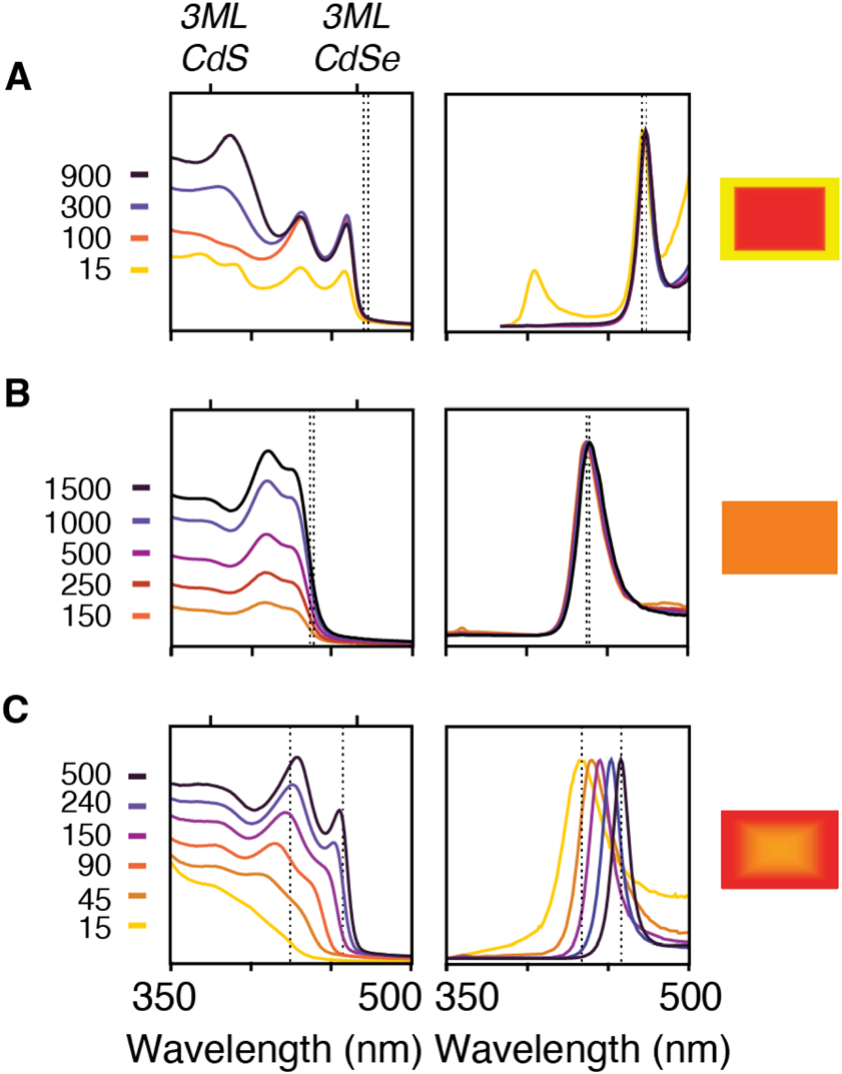
Absorption (left) and photoluminescence (center) from aliquots taken during the growth of (A) CdSe/CdS core crown prepared from 8 and 15 (B) CdSe_0.50_S_0.50_ alloy nanoplatelet prepared from 8 and 18 (C) CdSe_.50_S_.50_/CdSe core crown prepared from 3 and 18. The times indicated in the legend are in units of seconds. Cartoon nanoplatelet illustration (right) indicates the predicted nanoplatelet composition with the percent yellow and red indicating the percent CdS and CdSe, respectively. Dotted lines indicate the position of the luminescence maximum for each aliquot and illustrate the stokes shift and magnitude of the evolution of the band edge resonance in each synthesis.

For example, phase segregated CdSe/CdS core/crown nanoplatelets form when combining precursors with >10× relative *k*_obs_ (Fig. 4A). At early times the absorption spectrum of a relatively pure phase CdSe core nanoplatelet is observed that slowly adds spectral features of the CdS crown material. In these cases, the energy and width of the photoluminescence spectrum is similar to what is observed from pure phase materials.^34^

The growth of alloyed compositions, however, display broadened spectra that are shifted relative to the spectra of pure phase materials. By combining precursors with nearly matched conversion reactivities, homogeneous solid solutions can be synthesized, which were verified with powder X-ray diffraction (Fig. S14†). Fig. 4B displays the temporal evolution of aliquots during the formation of a homogeneous solid solution (CdS_1−*x*_Se_*x*_, *X* = 0.5) produced from 8 and 18 whose conversion reactivity differs 2×. Absent any competitive inhibition between precursors or attachment kinetics that are selective for one of the chalcogenides, identical conversion reactivity results in a S/Se solute composition set by the initial ratio of the precursors. The slow increase in intensity and lack of spectral shifting throughout the course of the nanoplatelet growth is consistent with this picture. This evolution supports the formation of a homogeneous composition that is relatively stable to ripening and phase segregation. STEM EELS mapping of Cd, Se, and S support a homogeneous distribution of these elements across the nanoplatelet (Fig. S15†). Similar results were obtained with several pairs of precursors with matched reactivity (see ESI†).

**Fig. 5:**
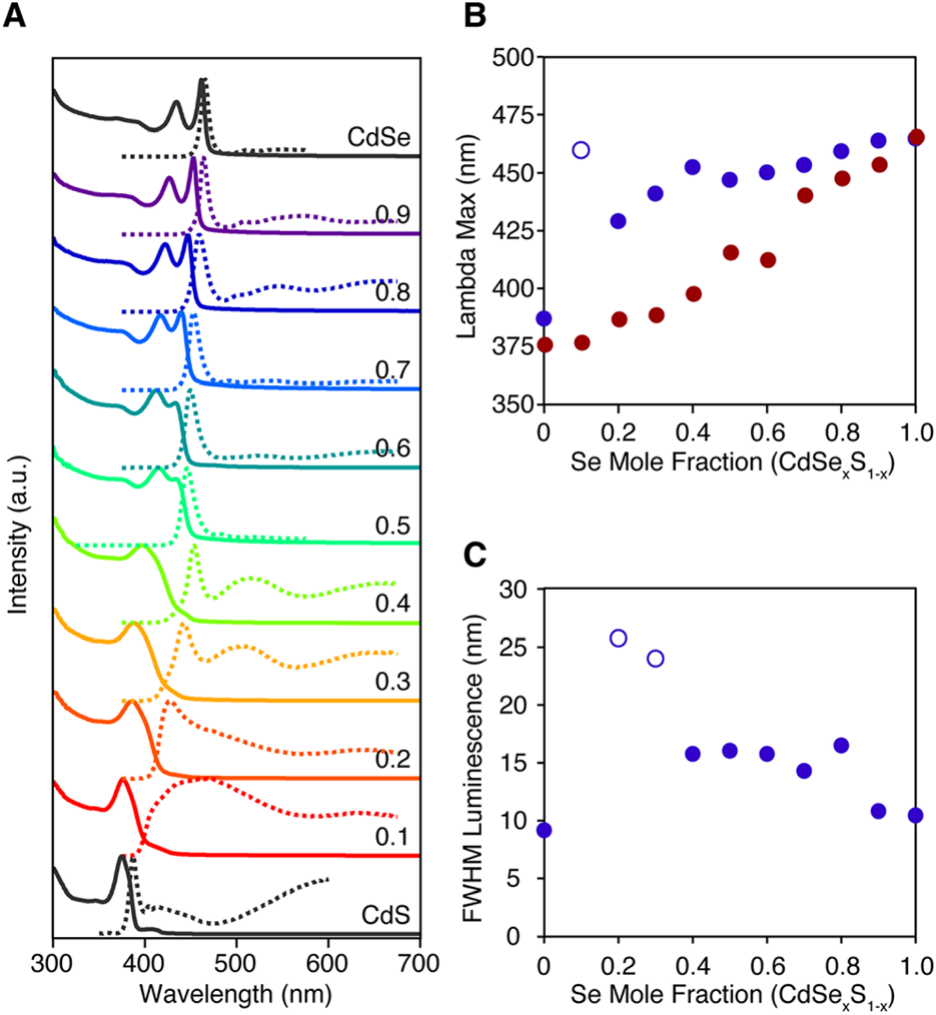
(A) Absorption (solid) and photoluminescence (dashed) spectra of homogeneously alloyed nanoplatelets prepared from 8 and 18. (B) The *λ*_max_ of the band edge absorption feature (red), the band edge luminescence (blue), and (C) the FWHM of the band edge luminescence feature were extracted using the multi-fitting function in Igor Pro and are plotted *versus* the mole fraction of the selenium. Open circles indicate spectra whose band edge luminescence could not be deconvoluted from broad self-trapped emission.

The luminescence from homogeneous alloy nanoplatelets occurs near the tail of the absorption onset, particularly in CdSe poor alloys (Fig. 5). These characteristics are distinct from pure phase and core/shell and core/crown architectures where the Stokes shift and linewidths are known to be much smaller.^35^ The spectral width of the band edge luminescence from alloyed nanoplatelets is also nearly twice that of pure phase CdSe or CdSe/CdS core/crown and core/shell microstructures (Fig. 5C).^23,36^ The relatively large breadth and apparent Stokes shift is to be expected from fluctuations of the alloy microstructure, and the increased electron phonon coupling caused by localization of excitons on impurities ions (*e.g.* Se substitution in CdS).^37^ Such effects are most significant in selenium poor alloys, where the FWHM and the apparent Stokes shift are greatest (Fig. 5B and C). The photoluminescence may be further broadened by heterogeneity in the distribution of nanoplatelet microstructures.

Pairs of sulfur and selenium precursors whose reactivity is <10× result in graded heterostructures. The phase segregation or extent of gradation from core to shell depends on the similarity of the *k*_obs_ and the ratio of precursors injected. Fig. 4C shows the spectral evolution of a graded CdS/CdSe nanoplatelet during growth. The simulated composition evolves from a mixed CdS_1−*x*_Se_*x*_ core rich in sulfur to a nearly pure phase CdSe region, which is consistent with the observed spectral shifting and narrowing of the absorption and photoluminescence linewidths. These changes are consistent with the localization of excitons to CdSe rich regions at nanoplatelet edges.

**Fig. 6:**
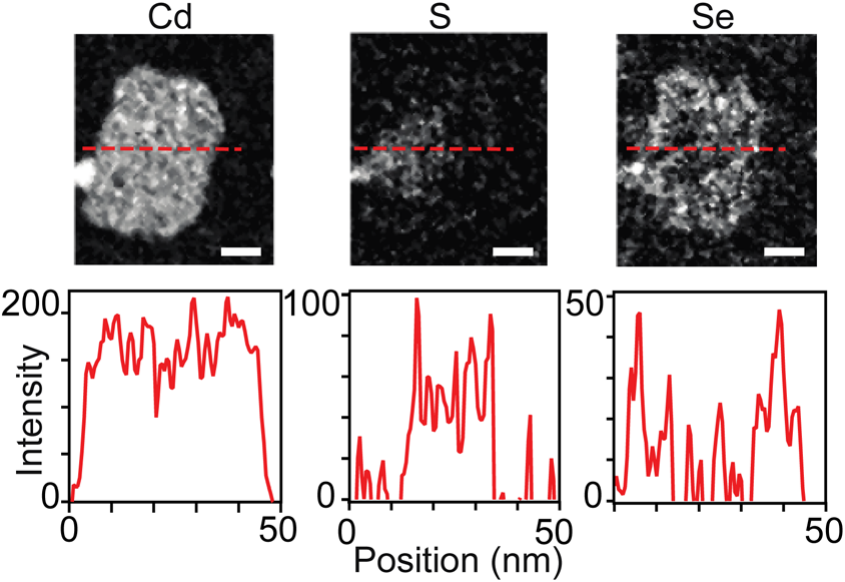
Elemental analysis of graded CdS/CdSe core/crown nanoplatelets using STEM EELS at 100 K. Cd, S, and Se maps with corresponding linescans (dashed red line above) below. The width of the scale bar corresponds to 10 nm.

While sulfur and selenium rich regions of 4 ML core–crown nanoplatelets can be imaged using transmission electron microscopy and energy dispersive X-ray spectroscopy (TEM-EDX),^38^ 3 ML nanoplatelets prepared in this study were unstable to beam damage unless measurements were conducted at 100 K or lower. At low temperatures it was possible to distinguish sulfide rich and selenide rich regions using scanning transmission electron microscopy and electron energy loss spectroscopy (STEM-EELS) (Fig. 6 and S16–S19†). However, the signal to noise was not sufficient to study the gradation in detail. Fig. 6 shows STEM-EELS 2D maps and linescans of a CdS_1−*x*_Se_*x*_/CdSe graded core/crown nanoplatelet. The distribution of selenium and sulfur is consistent with the spectral evolution. However, the signal to noise was insufficient to measure the slope of the gradation between CdS and CdSe in graded alloys and prevented more detailed analysis of the microstructure.

### Vibrational spectroscopy

**Fig. 7:**
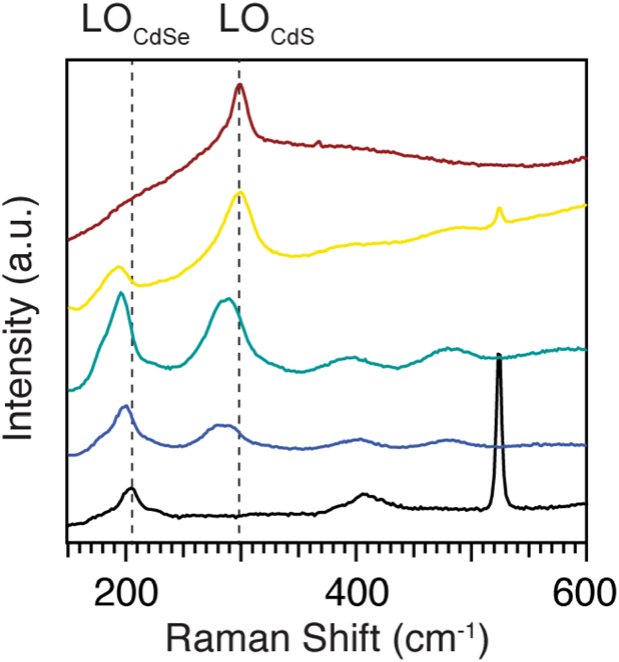
77 K Raman spectra of homogeneous alloys CdS_1−*x*_Se_*x*_, *x* = 0 (red), 0.2 (yellow), 0.5 (green), 0.7 (blue), 1(black). Dotted lines are guides that mark the LO frequencies of the pure phase nanoplatelets.

Fig. 7 shows Raman spectra of homogeneous alloys with compositions smoothly varying from pure CdSe to pure CdS. Fitting the Raman spectra of pure and heterostructured CdSe and CdS nanoplatelets shows there are two peaks near the Cd–Se stretching region at ∼200 cm^−1^ and two others near the Cd–S stretching region at ∼300 cm^−1^ (Fig. S10†). In each case, the lower energy peak is assigned to a surface optical (SO) mode and the higher energy peak to the longitudinal optical (LO) mode. We focus here on the stronger LO mode (*ν*(CdE)_LO_, *E* = S or Se) which can be seen in Fig. 7. The *ν*(CdSe)_LO_ frequency in pure CdSe is 203.7 cm^−1^, while for pure CdS the *ν*(CdS)_LO_ frequency is 298.8 cm^−1^. These peak frequencies are lower than the corresponding bulk values of 211 cm^−1^ for *ν*(CdSe)_LO_ and 305 cm^−1^ for *ν*(CdS)_LO_.^39^ The downward shift is caused by phonon confinement in the thickness direction.^40^ Vibrations perpendicular to the plane of the nanoplatelet are preferentially excited by the resonant excitation used here, enhancing their contribution to *ν*(CdE)_LO_.

However, our Raman measurements were performed at 77 K where anharmonic phonon–phonon coupling shifts these bands to higher frequencies.^41^ This effect can explain why our low temperature measurement is ∼4 cm^−1^ greater than a similar measurement conducted at room temperature (*ν*(CdSe)_LO_ (298 K) 199.5 cm^−1^).^40^ The magnitude of this temperature dependent shift is similar to previous studies on 5 ML CdSe nanoplatelets.^42^

In the Raman spectra of homogeneous alloys (CdS_1−*x*_Se_*x*_, *x* = 0, 0.2, 0.5, 0.7, and 1), both the *ν*(CdSe)_LO_ and *ν*(CdS)_LO_ frequencies decrease as their mole fraction decreases (Fig. 7 and S8†). The *ν*(CdSe)_LO_ shifts from 203.7 cm^−1^ for pure CdSe, to 194.9 cm^−1^ for CdS_0.8_Se_0.2_, and the *ν*(CdS)_LO_ shifts from 298.8 cm^−1^ for pure CdS to 292.6 cm^−1^ for CdS_0.3_Se_0.7_. The frequency changes induced by the alloy variation are consistent with the two mode behavior of bulk and quantum dot alloys of CdSe and CdS^11,43^ and strongly support the intermixing of sulfur and selenium on the atomic scale. We can compare these frequency shifts to spherical quantum dots composed of homogeneous CdS_1−*x*_Se_*x*_ alloys reported by us earlier.^11^ Those *ν*(CdSe)_LO_ frequencies are 2–5 cm^−1^ higher than in nanoplatelets of comparable composition. We attribute the lower nanoplatelet values to the stronger phonon confinement in the ∼0.9 nm thickness direction. In contrast, the quantum dot *ν*(CdS)_LO_ frequencies are 5–16 cm^−1^ lower than in nanoplatelets of comparable composition, suggesting that Cd–S stretches in alloyed nanoplatelets have more in-plane character than Cd–Se stretches.

Core–crown nanoplatelets whose interfaces have graded compositions display a *ν*(CdSe)^LO^ that shifts from 199.9 cm^−1^ to 196.7 cm^−1^ to 196.1 cm^−1^ as the interface becomes more diffuse: CdS/CdSe to CdS/CdS_1−*x*_Se_*x*_/CdSe to CdS_0.5_Se_0.5_ (Fig. S7†). The corresponding shift in *ν*(CdS)^LO^ is also to lower frequency: from 298.1 cm^−1^ to 294.7 cm^−1^ to 294.2 cm^−1^. For both LO modes, the phase segregated core/crown frequency is distinct from the graded alloy and homogeneous alloy, but the alloy structures (*i.e.* graded and homogeneous) cannot be distinguished on the basis of *ν*(CdE)_LO_ alone. Interestingly, for CdS/CdSe core/crown nanoplatelets, *ν*(CdS)^LO^ matches the frequency of the pure CdS nanoplatelet sample, while *ν*(CdSe)^LO^ is shifted to lower frequency relative to the pure material. Strain plays a role in heterostructure phonon frequencies. Past experiments on CdSe/CdS core/shell quantum dots show that when CdS has the dominant impact on the lattice constant for CdSe, the CdSe LO frequency will increase.^44^ As a result, our observations indicate that confinement has a larger effect on *ν*(CdSe)^LO^ than strain, possibly due to the relatively narrow crown region.

## Methods

### General considerations

All manipulations were performed in air unless otherwise indicated. Toluene (99.5%), hexanes (98.5%), methanol (99.8%), ethanol (≥99.8%), dichloromethane (≥99.5%), chloroform (≥99.8%), acetone (≥99.8%), acetonitrile (99.5%), oleic acid (99.99%) and 1-octadecene (90%), hexadecane (99%), tetraethylene glycol dimethyl ether (“tetraglyme” ≥99%), cadmium acetate dihydrate were obtained from either Sigma Aldrich and used without further purification. Substituted thioureas, thiones, and selones were prepared as reported previously. Synthesis and characterization of precursors shown in Table 1 was performed as described previously: (1, 4, 8);^30^ (2, 7);^25^3;^45^ (12–20).^11^ Compounds 5, 6, 9, and 10 were synthesized in similar manner and their NMR parameters described in the ESI.† Commercially available 2-imidazolidine thione (compound 11) was purchased from Sigma Aldrich and recrystallized prior to use.

UV-visible absorbance spectra were obtained using a PerkinElmer Lambda 950 spectrophotometer equipped with deuterium and halogen lamps. Photoluminescence measurements were performed using a Fluoromax 4 from Horiba Scientific.

### Synthesis of 3 ML nanoplatelets

In a nitrogen filled glovebox, stock solutions of thio- or selenourea precursors (0.1875 mmoles) are prepared in tetraglyme (1.25 mL, 150 mM solution). For heterostructured nanoplatelets, separate stock solutions were made but then combined in a single syringe in the desired ratio, stoppered with a rubber cap and removed from the glovebox. Precursors that do not readily dissolve at this concentration were removed from the glovebox in septum capped vials and pierced with an Argon inlet. Warming these solutions with an oil bath or heat gun caused the precursor to dissolve. The warm solution is then withdrawn using a syringe immediately before injection into the reaction vessel described below.

Ground cadmium acetate dihydrate (0.250 g, 1.08 mmol, 6.25 eq.) and 1-octadecene (14 mL) are added to a three neck round bottom flask equipped with a stir bar, a glass thermocouple well, and sealed with a septum. The flask is attached to a Schlenk line and degassed with vigorous stirring for 15 minutes before replacing the atmosphere with argon and heating the mixture to 195 °C. When the temperature reaches 100 °C, oleic acid (60.0 μL, 0.0537 g, 0.19 mmol) (1.26 eq.) of oleic acid are injected. When the temperature reaches 195 °C, the chalcogen precursor stock solution (1 mL) is immediately and swiftly injected. Aliquots for UV-vis absorption and photoluminescence measurements are removed throughout the reaction and diluted 35-fold with toluene.

After the reaction is complete, oleic acid (0.5 mL) is injected and the flask is removed from heat. When the reaction mixture has cooled under argon for approximately an hour, the mixture is cleaned by centrifuging at 7200 rpm for 3 minutes to remove any precipitate. The supernatant is diluted with acetone (10 mL) and the cloudy suspension centrifuged for 4 minutes. The nanoplatelet gel is then dispersed in toluene (5 mL) and acetone (10 mL) is added and the cloudy suspension centrifuged. The resulting nanoplatelets can then be suspended in toluene or hexanes (10 mL).

### Electron microscopy

Electron microscopy samples were prepared by further diluting reaction aliquots with toluene (4–5× that of the concentration used for UV-vis absorption). Nanoplatelets lie flat upon when dropcast on the dull side of Ted Pella # 10824 ultra-thin carbon film on lacey carbon support film grids.

Nanoplatelet imaging and sizing by STEM was performed on an FEI Talos F200X at 200 kV. STEM energy dispersive spectra were measured with a SuperX-EDS system using spectra mode. Elemental weight percentages were calculated from S_Kα_: 2.23–2.48 keV; Se_Lα,β_: 1.16–1.60 keV; Se_Kα_: 11.00–11.40 keV; Se_Kβ_: 12.36–12.68 keV; Cd_Lβ_: 3.00–3.65 keV. Detailed discussion of nanoplatelet dimension measurements and error is found in the ESI.†

STEM-EELS was performed on an aberration-corrected FEI Titan Themis 300 operating at 120 kV equipped with 965 GIF Quantum ER to access the cadmium M_4,5_ edge at 404 eV, the selenium L_2_ and L_3_ edges at 1476 and 1436 eV, and the sulfur L_2,3_ edge at 165 eV. The sulfur K edge at 2472 eV proved to be inaccessible. Spectra were recorded on either a Gatan K2 direct electron detector operating in electron counting mode or an UltraScan 1000 CCD detector with DualEELS recording to probe the sulfur L_2,3_ and selenium L_2,3_ edge simultaneously. DualEELS cycles between the high loss and low loss regions^46^ so that the maps can be overlaid (Fig. 4B). All data was collected at −140 °C to minimize radiation damage under the electron probe. Data was analyzed using Cornell Spectrum Imager Toolkit in FIJI/ImageJ.^47^

### Raman spectroscopy measurements

Raman spectroscopy measurements are performed on a home built micro-Raman spectrometer. Nanoplatelet samples are drop cast onto silicon wafers and loaded into a Cryo Industries optical cryostat and cooled to 77 K with liquid nitrogen. At 77 K the signal-to-noise is significantly improved for these samples (Fig. S9†). An Ondax laser (*λ* = 406 nm) enters a Nikon Ti/U inverted microscope, reflects off a beamsplitter (70% T/30% R), and is focused onto the sample by an objective (40×/0.6NA). The backscattered light is collected by the same objective and directed through a pinhole (50 μM), passed through two notch filters (*λ* = 405 nm), and focused into an Acton spectrometer (0.3 m) with an 1800 g mm^−1^ grating and dispersed onto a Princeton Instruments PIXIS-400 CCD array detector. Typical laser powers are 1 mW and typical acquisitions times are 150 s. We perform multiple consecutive measurements on the same location to check for sample degradation. Spectra are calibrated with an Argon calibration lamp and typical instrument resolution is 6 cm^−1^ as determined by the peak of the Argon lamp (*λ* = 415.9 nm).

We measured two 3 ML nanoplatelet series with Raman spectroscopy. In the first series, nanoplatelets are made with precursors with matched reactivities and varying Se : S ratios and thus constitute homogeneous alloys CdS_1−*x*_Se_*x*_, with *x* = 0, 0.2, 0.5, 0.7, and 1. In the second series, the composition ratio of selenium to sulfur of each nanoplatelet is held constant at 50% Se and 50% S, while the morphology of the interfacial region is varied: phase segregated core/crown nanoplatelets (CdS/CdSe), nanoplatelets with a more diffuse interfacial region (CdS/CdS_1−*x*_Se_*x*_/CdSe), and a homogeneous alloy (CdS_0.5_Se_0.5_). For all of the samples, other than pure CdS (*i.e.* CdS_1−*x*_Se_*x*_, with *x* = 0), the excitation laser is resonant with the lowest energy excitonic transitions or just above. All data were fit using Lorentzian line shapes and suggest fit uncertainties in the peak frequencies lower than ±1 cm^−1^, and measurement to measurement variability of the peak frequencies closer to ±1.5 cm^−1^.

## Conclusions

The results above support a homogeneous nucleation and growth mechanism that proceeds from a burst of nucleation followed by growth. Precursor conversion limits the kinetics under these conditions enabling the solute and the nanoplatelet microstructure to be controlled by the precursor reactivity. Moreover, the extent of nanoplatelet nucleation can be controlled by the precursors chosen. Phase segregated, graded, and homogeneous CdS_1−*x*_Se_*x*_ alloy compositions can thus be obtained with high fidelity. The observed structures and spectra indicate that growth from the mixed solute composition is relatively unselective for either the sulfide or selenide containing solutes.

## Author contributions

The manuscript was written through contributions of all authors. All authors have given approval to the final version of the manuscript. N. S., L. H., A. W., A. K., B. H. G., I. B., and A. C. C. performed experimental investigations and data analysis. The project was conceived by N. S., L. H., A. C. C., B. D., L. K., and J. O.

## Conflicts of interest

There are no conflicts to declare.

## Supplementary Material

SC-014-D3SC03384H-s001
