# Supplementary material for: Synthesis of graded CdS1−xSex nanoplatelet alloys and heterostructures from pairs of chalcogenoureas with tailored conversion reactivity
Source: Chem Sci. 2023 Oct 16;14(43):12345–54. doi: 10.1039/d3sc03384h (PMC10631235; doi:10.1039/d3sc03384h)
Supplement: SC-014-D3SC03384H-s001 [file SC-014-D3SC03384H-s001.pdf]

Electronic Supplementary Material (ESI) for Chemical Science.
This journal is © The Royal Society of Chemistry 2023

Supporting Information for

Synthesis of graded $CdS_{1-x}Se_x$ nanoplatelet alloys and heterostructures from pairs of chalcogenoureas with tailored conversion reactivity

Natalie Saenz, Leslie Hamachi, Anna Wolock, Berit Goodge, Alexis Kuntzmann, Benoit Bubertret, Isabel Billinge, Lena Kourkoutis, David Muller, Andrew C. Crowther, Jonathan S. Owen*

Department of Chemistry, Columbia University, New York, NY
Department of Chemistry, Barnard College, Columbia University, New York, NY
Kavli Institute for Electron Microscopy, APAM, Cornell University, Ithaca, NY

Table of Contents

1. Single exponential fits of kinetics data: CdS ………… 3
2. Single exponential fits of kinetics data: CdSe ………… 4
3. STEM Measurement Error Discussion and sizing examples ……… 5–7
4. Raman Spectra and Fitting Results ..…… 8–12
5. New precursors NMR spectra …… 13–14
6. STEM EELS Maps and linescans …… 15–19
7. Powder x-ray diffraction of alloys …… 20
8. References ..……… 21

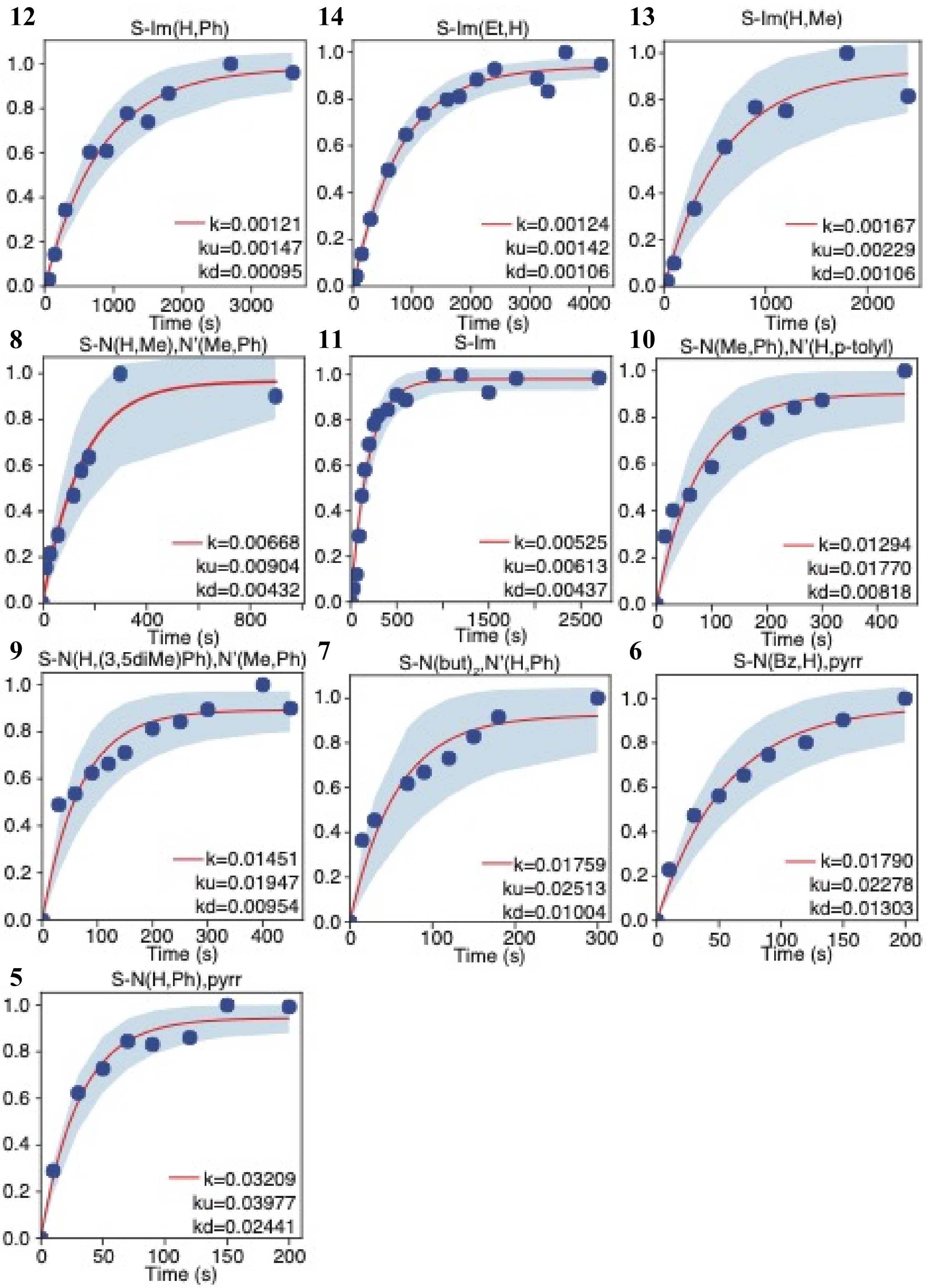

Figure S1. Kinetics of CdS formation fit to a single exponential. The y-axis is the normalized yield as measured using the absorption of aliquots. The light blue shaded region represents 2 standard deviations in the red curve's fit to the data, giving the upper (k_u) and lower (k_d) limits of the k_{obs} values.

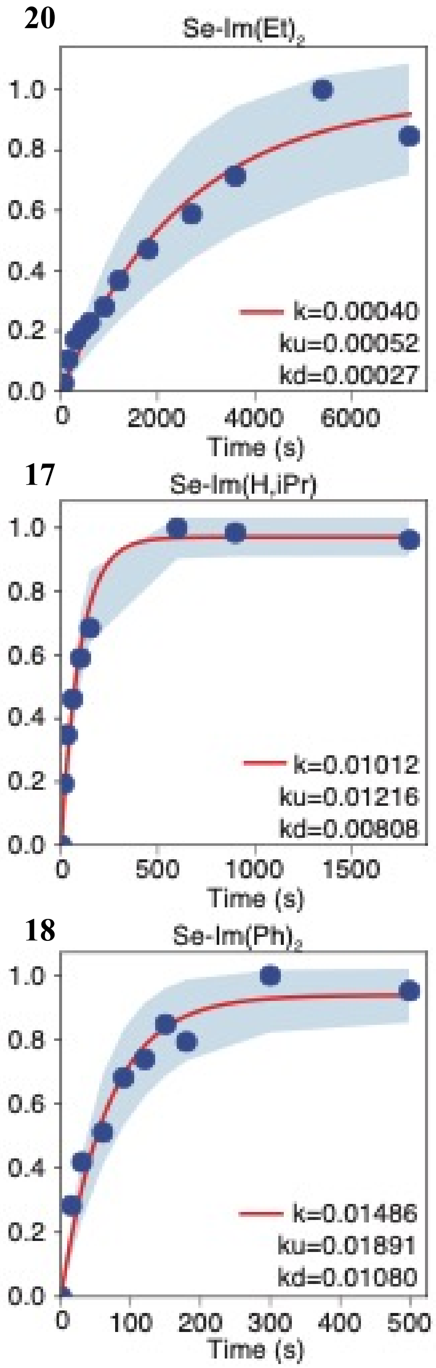

Figure S2. Kinetics of CdSe formation fit to a single exponential. The y-axis is the normalized yield as measured using the absorption of aliquots. The light blue shaded region represents 2 standard deviations in the red curve's fit to the data, giving the upper (k_u) and lower (k_d) limits of the k_{obs} values.

STEM Measurement Error Analysis. Nanoplatelet sizes were determined using high angle annular dark field scanning transmission electron microscopy (HAADF-STEM). There are a number of potential sources of error in the measurements: (1) The nanoplatelets produced by our synthetic method are not perfect rectangular prisms. (2) Few nanoplatelets lay perfectly flat. (3) Nanoplatelets with extremely large lateral dimensions roll up like scrolls to reduce strain[1, 2].

The final size of the nanoplatelets was measured using STEM analysis of an aliquot taken at the reaction endpoint. The reaction endpoint was understood as the time at which the absorbance at the band edge reached a relative maximum. This time generally corresponded to twice the half-life calculated from the reactivity coefficient ($t_{1/2} = 0.6931/k_{obs}$,). Choosing the appropriate aliquot for the final size of the nanoplatelet was done for each reaction individually and often several of the later aliquots were measured to validate a final size, confirming that the size plateaus at the endpoint.

Nanoplatelets may grow laterally beyond the end point, presumably via Ostwald ripening, but only if the reaction time greatly exceeds the reaction endpoint. Alternatively nanoplatelets with greater thickness can appear in the absorption spectra, which can cause the lateral dimensions to decrease. For this reason, measurements of the final nanoplatelet size was conducted with the final aliquot rather than the final reaction mixture. No washing procedure was used prior to HAADF-STEM analysis.

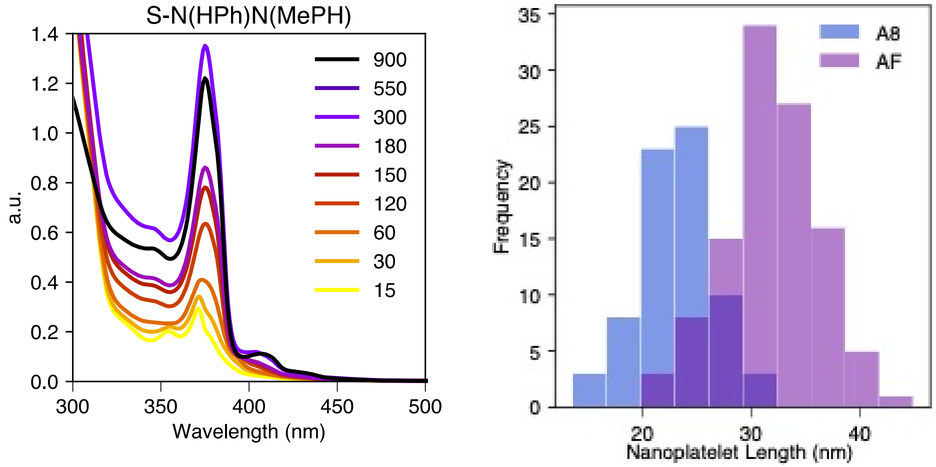

Figure S3. CdS nanoplatelets synthesized from **8**. Left: Absorbance of timed aliquoting. Right: Histogram of the nanoplatelet STEM length measurements taken of the aliquot at 550s (A8, blue) compared to the final, washed reaction mixture (AF, violet)

The final aliquot is diluted and dropcast onto lacey carbon coated copper TEM grids. Nanoplatelets tend to stack and the dilutions should balance a high enough concentration to obtain images of a sufficient number of nanoplatelets and a low enough concentration to avoid aggregated groups of nanoplatelets. Typically aliquots diluted for UV-vis analysis (35x dilution of a 0.1mL aliquot) were further diluted 4-7x prior to drop casting on TEM grids. The lengths of 70-100 nanoplatelets imaged by HAADF-STEM at various magnifications were measured (Figure S3, right). Nanoplatelets were measured if (1) the whole nanoplatelet was visible (2) it could be identified as a single nanoplatelet (3) it was not excessively rolled.

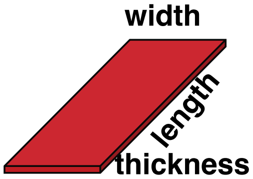

Here, we define "length" as the longer edge and "width" as the shorter edge, which is assumed to be the unrolled edge, as shown above. Figure S4 shows examples of nanoplatelets that were measured from a HAADF STEM image. The red lines indicate lengths used. Only nanoplatelets with clearly visible edge lengths were measured, out of many nanoplatelets within the field of view. Approximately 10–20 images with different magnifications were used to obtain an average. Only the largest CdSe nanoplatelets rolled into scrolls with regions that overlapped (one or more full rolls). In these two cases, we measured the width of a single roll and estimated the total width (circumference of the scroll) by multiplying by pi. Assuming our largest error is whether the scroll is rolled somewhere between one half to two times, the error in these is then ± half π times the roll diameter. Histograms of measurements are shown below.

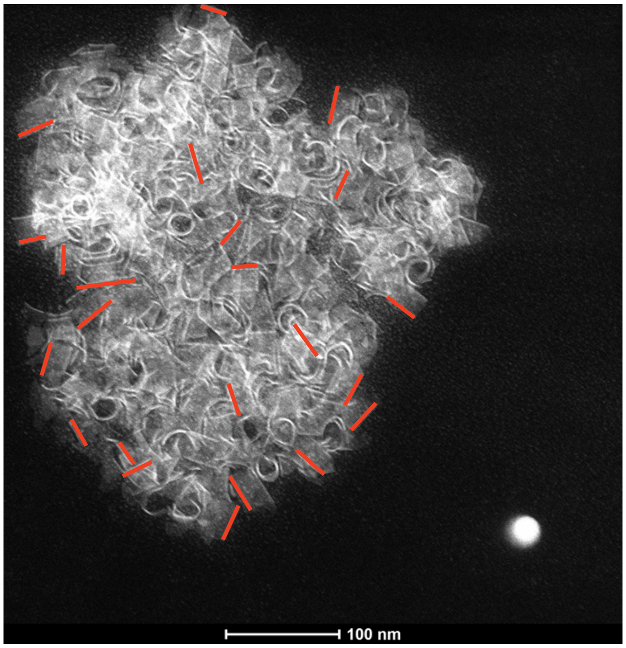

Figure S4. Precursor **15** (Notebook number: NSB-02-083B), 200kV, 225kx.

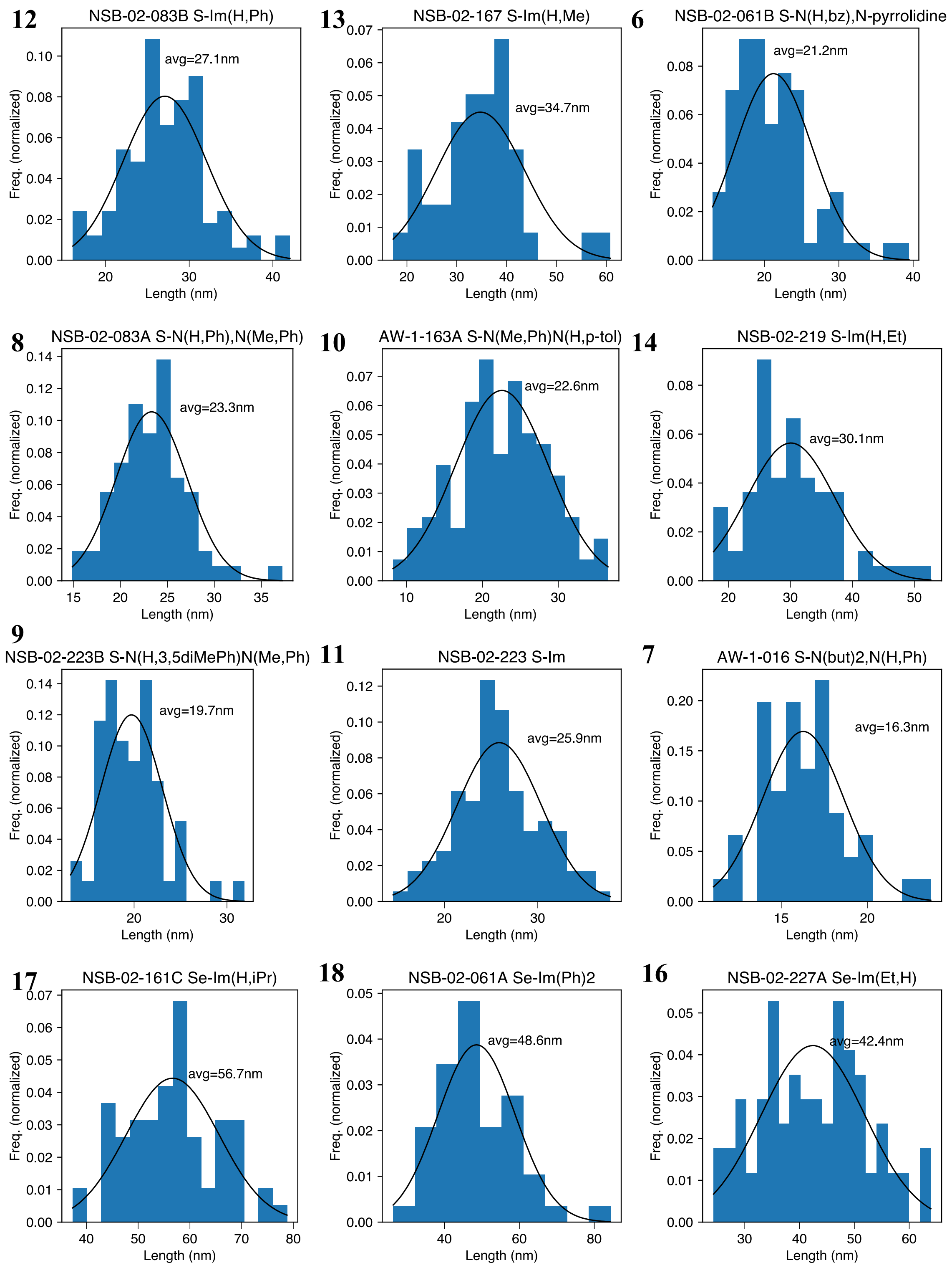

Figure S5. Sizing histograms produced using the sizing method described above for the precursor indicated in the upper left hand corner of each histogram.

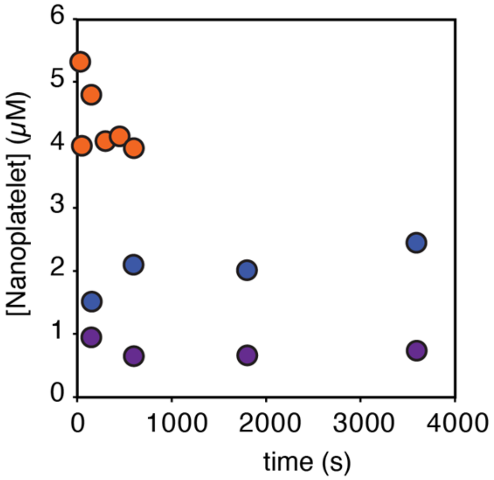

Figure S6. Nanoplatelet concentrations in aliquots as determined from the ratio of the yield as measured by UV-Vis absorption and from STEM analysis of the size in each aliquot. While the absolute error in the size shown in Figure 3 applies to these data, the relative error in the orange and blue data is small, as the size reaches a stable endpoint within the timeframe shown. Thus, the stability in the nanoparticle size at long times indicates that the concentration of nanoplatelets is stable to ripening. Purple - **20**, blue - **19**, orange - **18.**

Raman Spectroscopy.

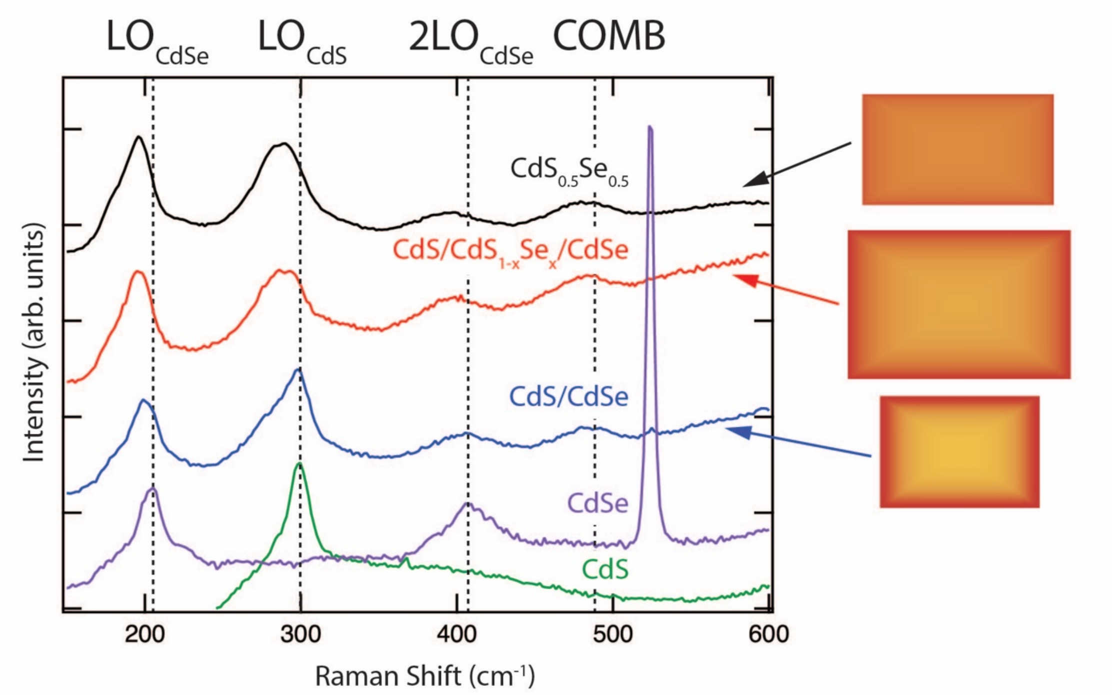

Figure S7. Raman spectra of various types of nanoplatelet heterostructures. From top to bottom: a homogeneous alloy, a CdS/CdSe core/crown with a graded alloy interface, a CdS/CdSe core/crown, pure CdSe, and pure CdS nanoplatelets. Dotted lines are guides that mark the LO and 2LO frequencies of the pure phase nanoplatelets and a combination band seen in the heterostructured nanoplatelets.

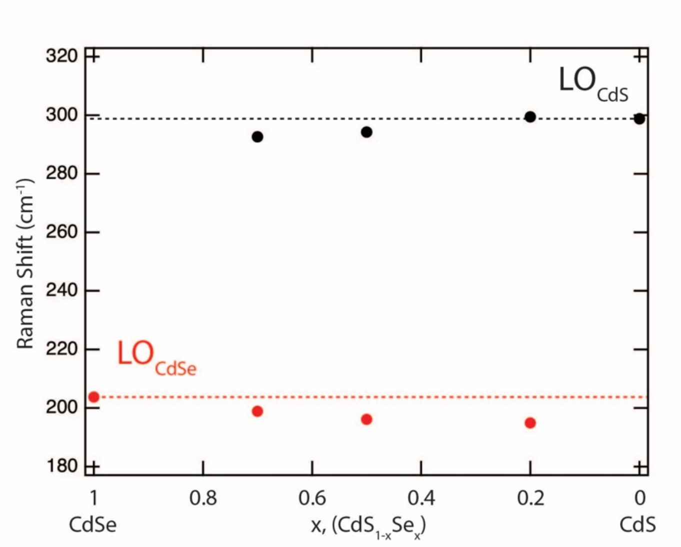

Figure S8: LO frequency for CdS and CdSe stretches as a function of selenium content for the alloy series CdSe, $CdS_{0.3}Se_{0.7}$, $CdS_{0.5}Se_{0.5}$, $CdS_{0.8}Se_{0.2}$, and CdS. Dashed lines are the LO frequencies for pure CdSe (red) and pure CdS (black) nanoplatelets. Uncertainties in these measurements are smaller than the data points (less than ± 1.5 cm^{-1})

Temperature-Dependent Raman Spectra. The nanoplatelet Raman spectra are weak at room temperature, with increasing peak intensities as the temperature drops (Figure S9). One possible explanation for this temperature trend is a shift in the electronic spectra with thermal contraction as the sample cools. As the nanoplatelet cools, it contracts, and the corresponding change in thickness causes a shift in electronic state energies for a different degree of quantum confinement. This shift can bring electronic states that are more strongly coupled to the LO phonons into resonance with the laser frequency, causing increased resonance enhancement at low temperatures.

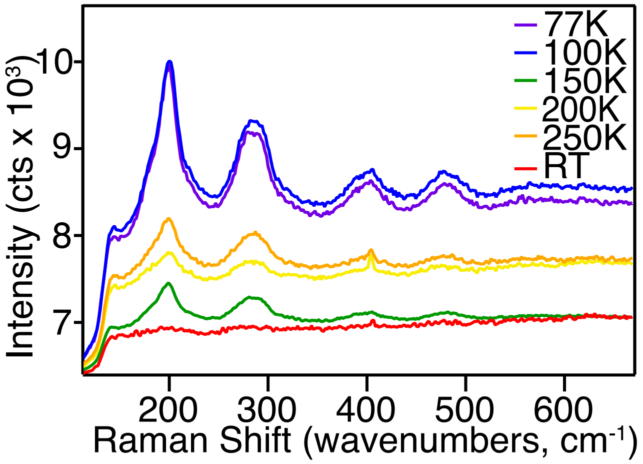

Figure S9. Raman spectra of $CdS_{0.3}Se_{0.7}$ at different temperatures. As temperatures increase, the signal-to-noise decreases.

Fitting Results for Raman Spectra of Nanoplatelets.

Raman spectra are fit to Lorentzians. In general, the CdSe stretch peak at ~200 cm^{-1} and the CdSe stretch peak at ~300 cm^{-1} each fit to two peaks, a lower frequency surface optical mode, and a higher energy longitudinal optical mode. For pure CdS nanoplatelets, we fit the CdS stretch peak to a single Lorentzian due to a steeply sloped baseline. The CdSe stretch peak for pure CdSe fit best to three peaks, with an additional peak at slightly higher energy to the LO mode. Raman spectra are scaled to produce similar peak intensities for plotting purposes.

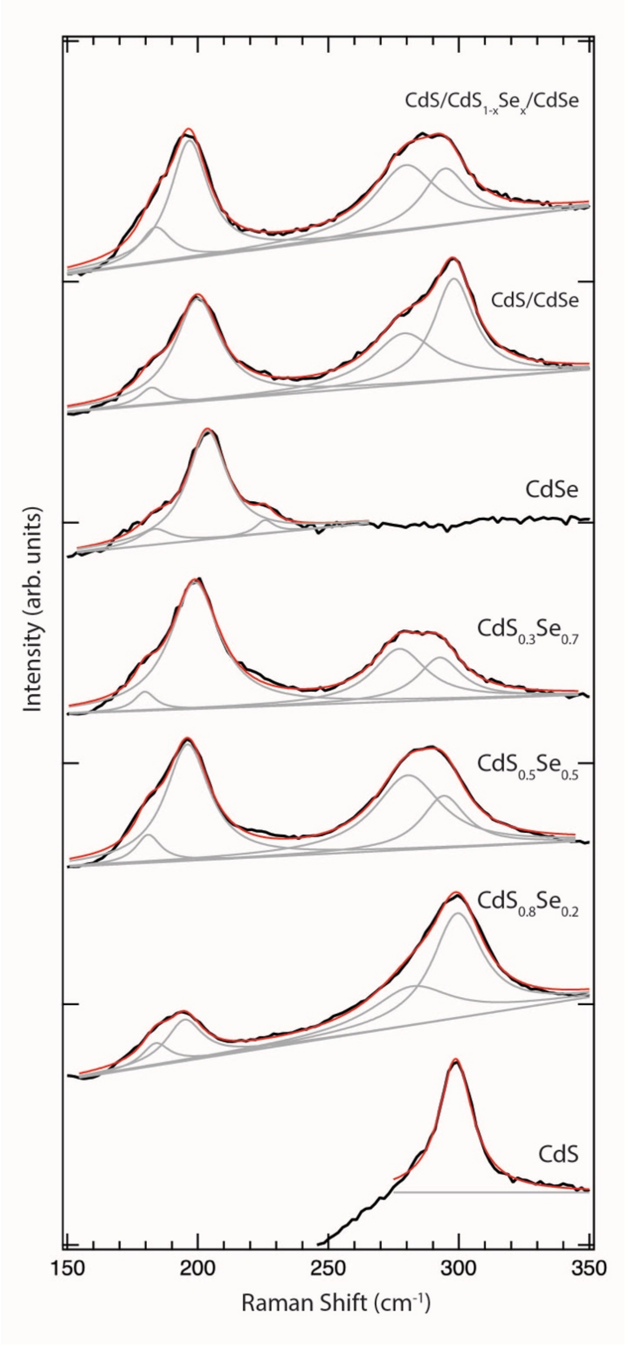

Figure S10. Lorentzian fits to Raman spectra for each nanoplatelet sample. The Raman spectra, total fit, and individual peak fits are black, red, and gray, respectively.

Table S1: CdSe Surface Optical Phonon Fit Parameters

Sample	ω (cm^{-1})	Height	Area (cm^{-1})	FWHM (cm^{-1})
Pure CdS	N/A	N/A	N/A	N/A
$CdS_{0.8}Se_{0.2}$	183.9 ± 1.2	0.19 ± 0.04	3.7 ± 1.7	12.8 ± 3.4
$CdS_{0.5}Se_{0.5}$	181.0 ± 0.6	0.23 ± 0.03	4.2 ± 1.1	11.6 ± 2.3
$CdS_{0.3}Se_{0.7}$	179.7 ± 0.7	0.16 ± 0.02	2.5 ± 0.7	9.9 ± 2.6
Pure CdSe	183.4 ± 1.3	0.13 ± 0.02	3.0 ± 1.0	14.8 ± 4.4
$CdS/CdS_{1-x}Se_x/CdSe$	183.8 ± 1.0	0.30 ± 0.04	7.1 ± 2.0	15.2 ± 2.7
CdS/CdSe	182.3 ± 0.9	0.15 ± 0.02	2.6 ± 0.8	11.5 ± 3.2

Table S2: CdSe Longitudinal Optical Phonon Fit Parameters

Sample	ω (cm^{-1})	Height	Area (cm^{-1})	FWHM (cm^{-1})
Pure CdS	N/A	N/A	N/A	N/A
$CdS_{0.8}Se_{0.2}$	194.9 ± 1.0	0.34 ± 0.04	9.2 ± 1.8	16.9 ± 2.1
$CdS_{0.5}Se_{0.5}$	196.1 ± 0.3	0.97 ± 0.02	31.2 ± 1.4	20.5 ± 0.8
$CdS_{0.3}Se_{0.7}$	198.9 ± 0.2	1.05 ± 0.01	37.7 ± 1.0	22.8 ± 0.6
Pure CdSe	203.7 ± 0.2	0.90 ± 0.02	25.8 ± 1.3	18.2 ± 0.9
$CdS/CdS_{1-x}Se_x/CdSe$	196.7 ± 0.4	0.98 ± 0.04	27.3 ± 2.1	17.7 ± 0.9
CdS/CdSe	199.9 ± 0.3	0.85 ± 0.02	28.4 ± 1.1	21.1 ± 0.8

Table S3: CdS Surface Optical Phonon Fit Parameters

Sample	ω (cm^{-1})	Height	Area (cm^{-1})	FWHM (cm^{-1})
Pure CdS		Not Observed in Fit		
$CdS_{0.8}Se_{0.2}$	281.9 ± 2.6	0.32 ± 0.05	18.8 ± 4.4	37.2 ± 3.4
$CdS_{0.5}Se_{0.5}$	280.5 ± 1.6	0.62 ± 0.09	30.1 ± 5.6	31.0 ± 1.9
$CdS_{0.3}Se_{0.7}$	277.2 ± 1.2	0.44 ± 0.05	17.1 ± 3.0	24.9 ± 2.3
Pure CdSe	N/A	N/A	N/A	N/A
$CdS/CdS_{1-x}Se_x/CdSe$	279.6 ± 1.6	0.54 ± 0.07	24.9 ± 4.8	29.3 ± 2.4
CdS/CdSe	279.0 ± 1.1	0.43 ± 0.03	20.0 ± 2.4	29.9 ± 2.3

Table S4: CdS Longitudinal Optical Phonon Fit Parameters

Sample	ω (cm^{-1})	Height	Area (cm^{-1})	FWHM (cm^{-1})
Pure CdS	298.8 ± 0.1	1.11 ± 0.01	27.2 ± 0.6	15.6 ± 0.4
$CdS_{0.8}Se_{0.2}$	299.4 ± 0.4	0.87 ± 0.06	31.6 ± 3.9	23.1 ± 1.4
$CdS_{0.5}Se_{0.5}$	294.2 ± 1.1	0.4 ± 0.1	14.1 ± 5.2	20.7 ± 3.2
$CdS_{0.3}Se_{0.7}$	292.6 ± 1.1	0.35 ± 0.05	11.3 ± 2.8	20.4 ± 2.7
Pure CdSe	N/A	N/A	N/A	N/A
$CdS/CdS_{1-x}Se_x/CdSe$	294.7 ± 1.0	0.47 ± 0.08	15.8 ± 4.5	21.3 ± 2.8
CdS/CdSe	298.1 ± 0.3	0.85 ± 0.03	25.4 ± 2.0	19.1 ± 1.0

Table S5: Pure CdSe 3rd Peak

Sample	ω (cm^{-1})	Height	Area (cm^{-1})	FWHM (cm^{-1})
Pure CdSe	225.7 ± 1.0	0.11 ± 0.02	1.5 ± 0.5	8.5 ± 3.3

^{1}H NMR Spectrum of 5.

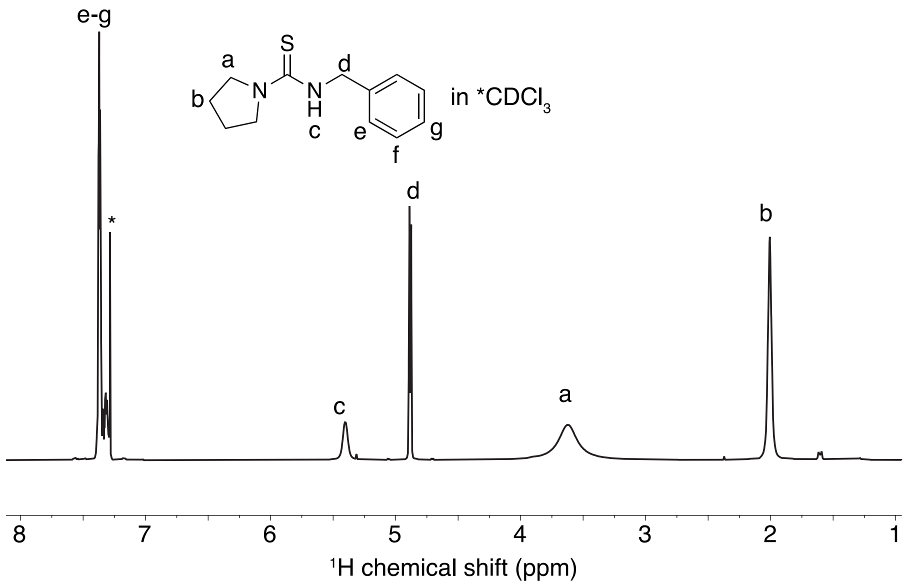

^{1}H NMR Spectrum of 6.

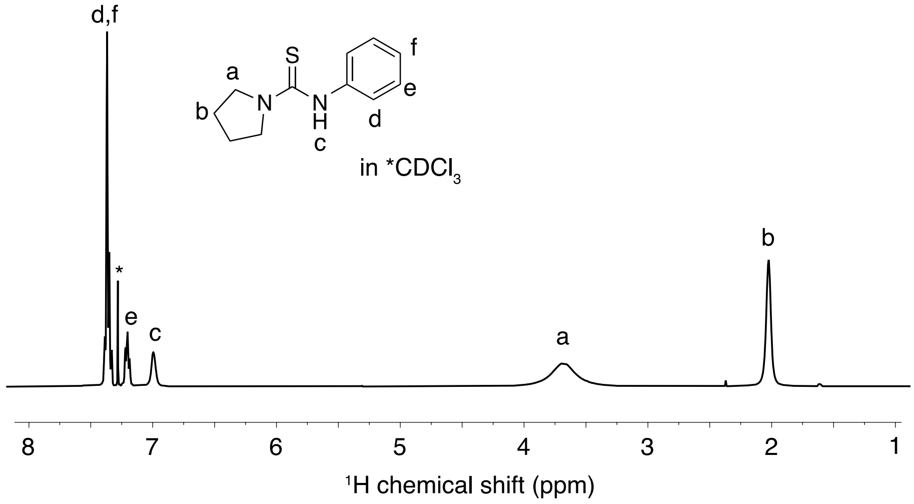

^{1}H NMR Spectrum of 9.

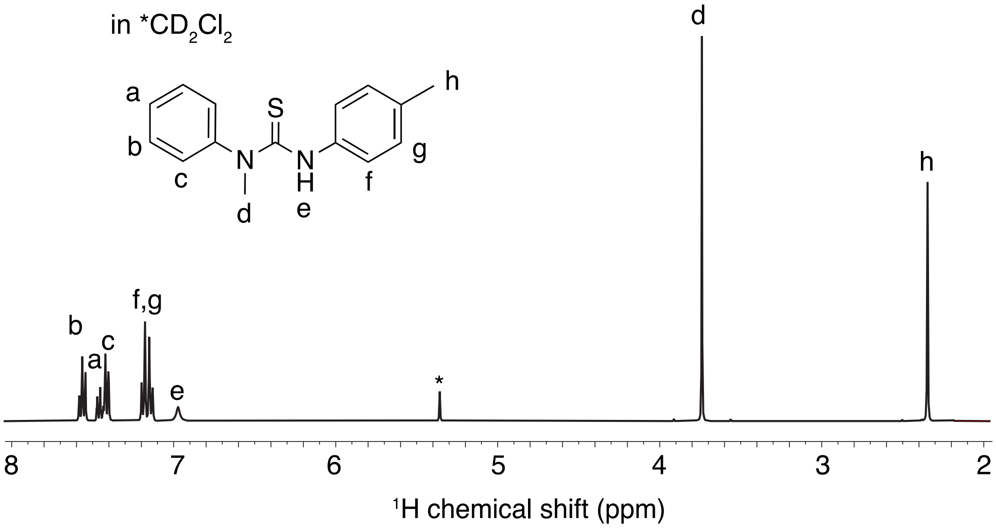

^{1}H NMR Spectrum of 10.

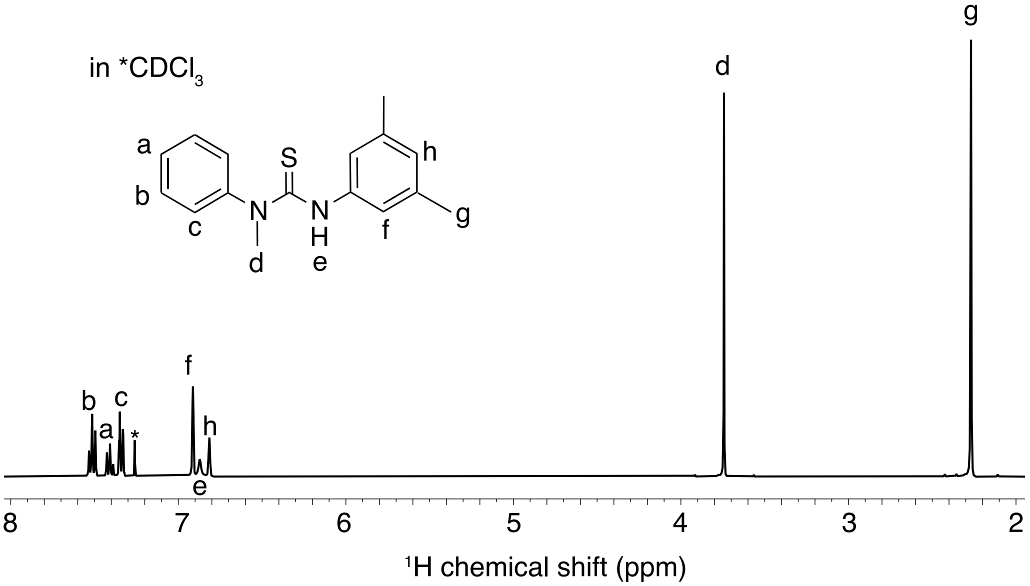

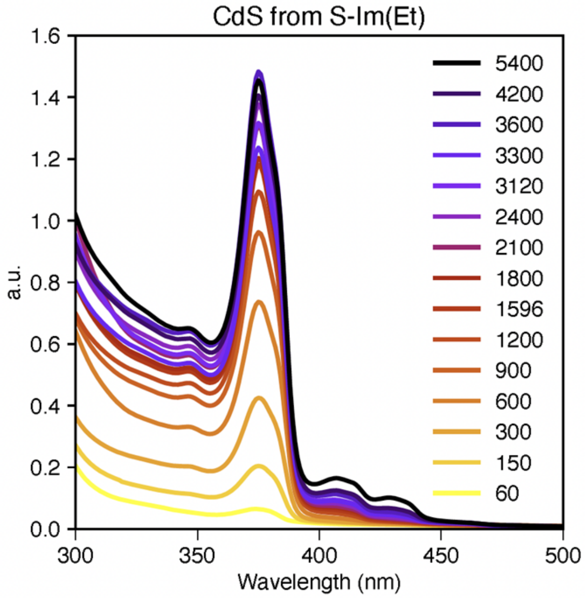

Figure S11. Quantitative absorption spectra of CdS Nanoplatelets made from **14**. 4ML and 5ML nanoplatelets form after the reaction's endpoint (1200s). Each trace corresponds to a different time point indicated in the legend with units of seconds.

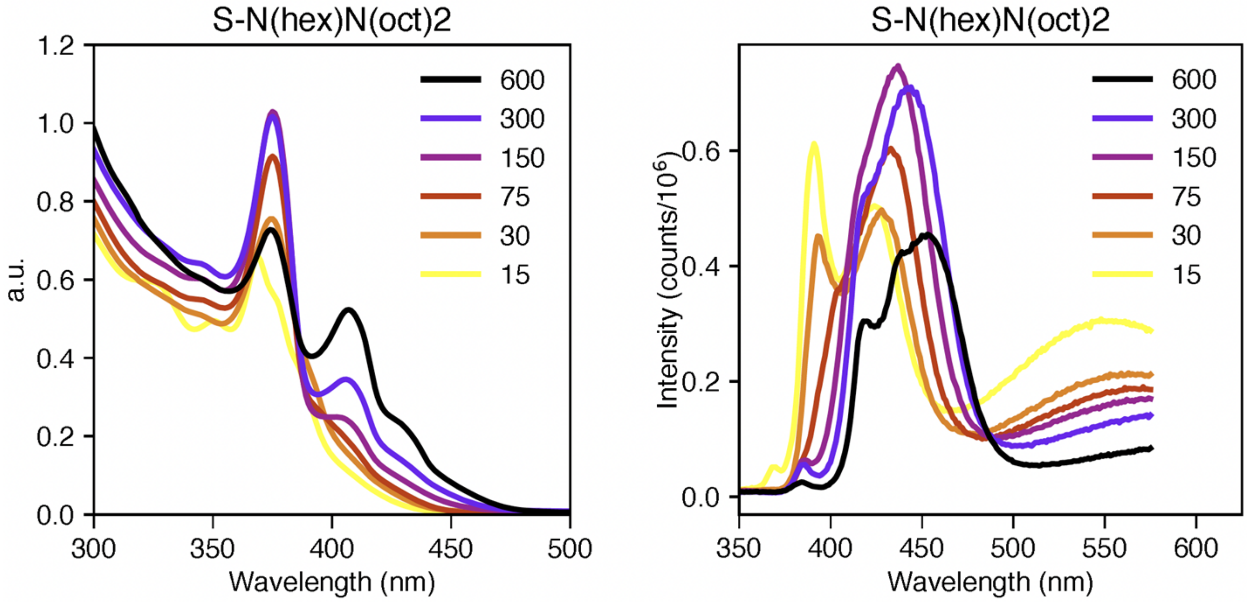

Figure S12. Quantitative absorption (left) and luminescence (right) spectra of CdS Nanoplatelets made from **2**. 4ML and 5ML nanoplatelets form after the reaction's endpoint (600s respectively). Each trace corresponds to a different time point indicated in the legend with units of seconds.

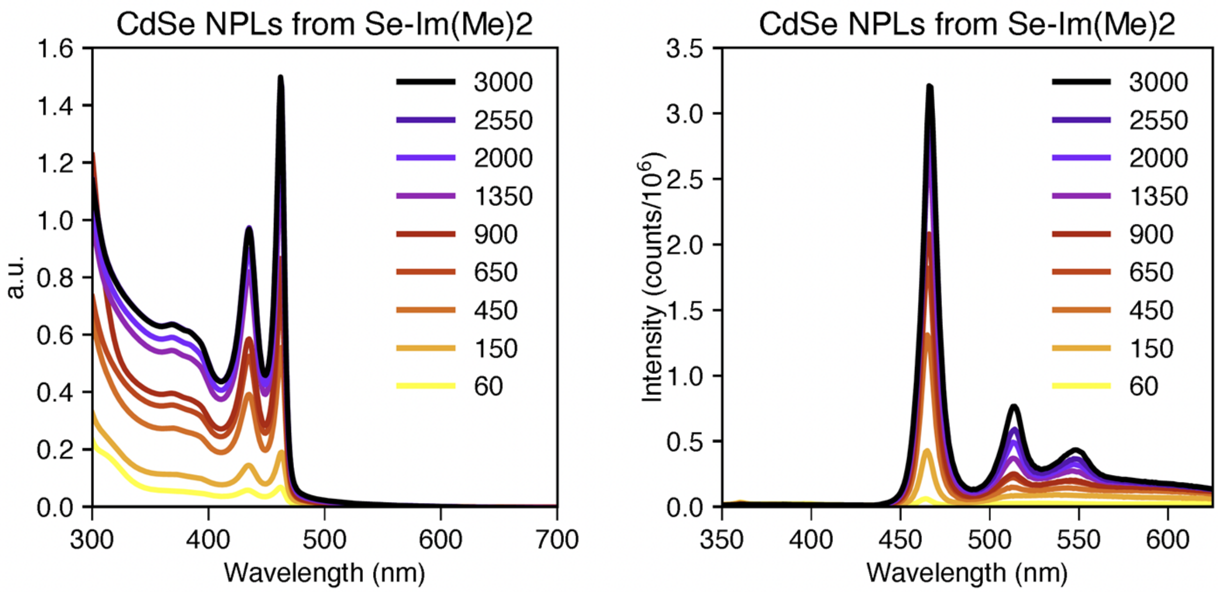

Figure S13. Quantitative absorption (left) and luminescence (right) spectra of CdSe prepared from **19**. 4ML and 5ML nanoplatelets form at long reaction times. Each trace corresponds to a different time point indicated in the legend with units of seconds.

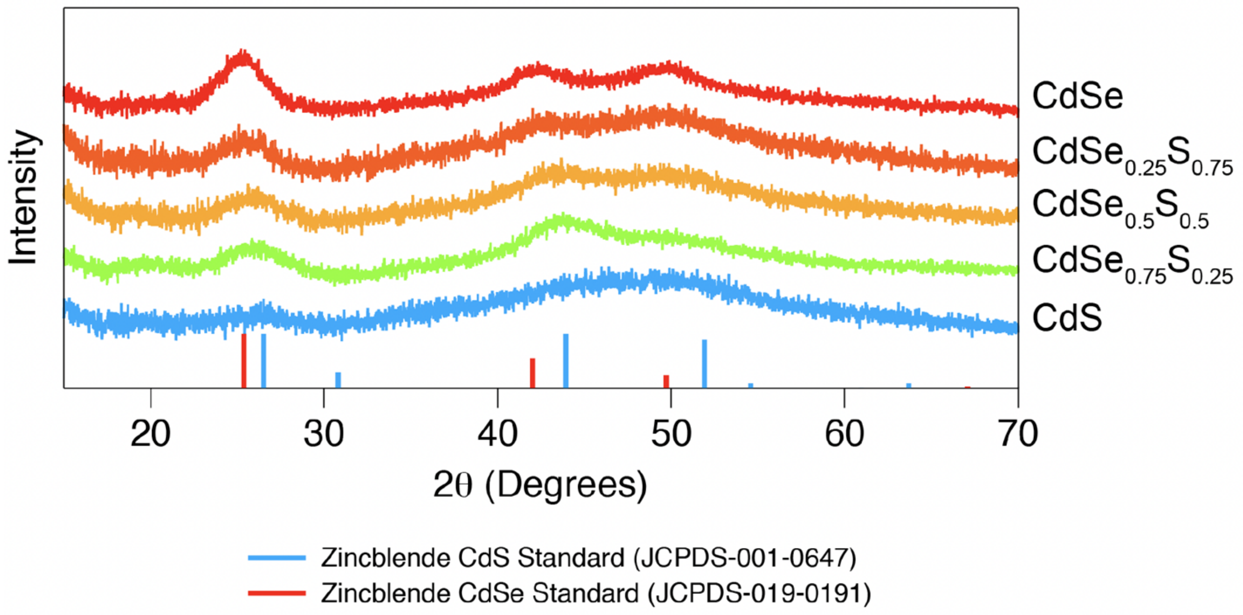

Figure S14. Powder x-ray diffraction of homogeneous alloyed nanoplatelets illustrate the shift from low to high angle as the composition varies from CdSe to CdS. The breadth and position of the diffraction peaks is a result of the 3ML thickness and may be influenced by the extent of nanoplatelet rolling.

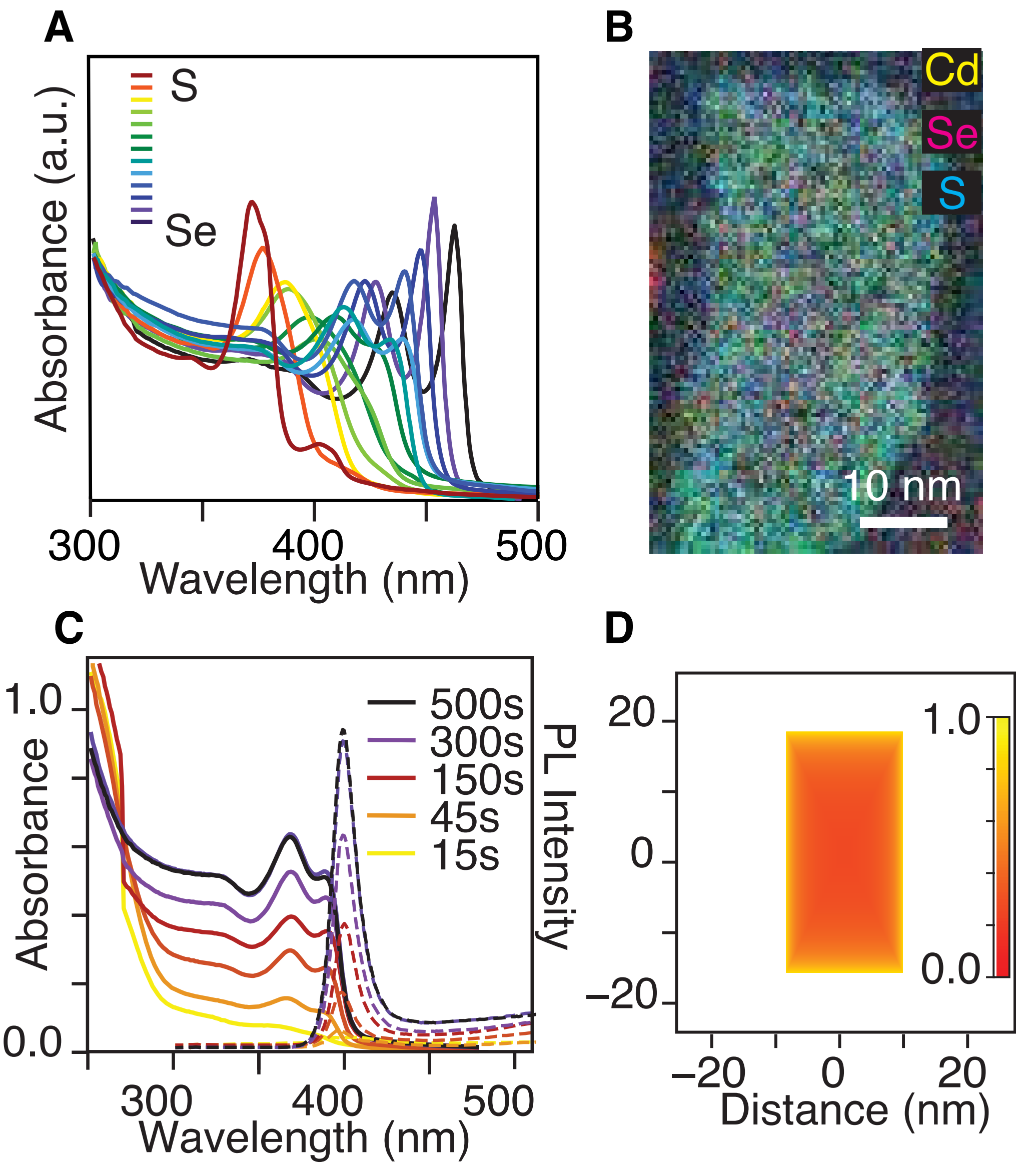

Figure S15. (A) Normalized absorption spectra of homogeneous alloyd nanoplatelets as the concentration changes from pure phase CdS (red) to pure phase CdSe (black). The ratios are x=1.0, x=0.90, x=0.80, x=0.70, x=0.66, x=0.60, x=0.50, x=0.40, x=0.33, x=0.30, x=0.20, x=0.10, x=0 (B) Dual-EELS of a CdSe.50/CdS.50 nanoplatelets Cd: yellow, S: cyan, Se: magenta showing a homogeneous distribution of elements. (C) Absorbance and emission of CdSe.50/CdS.50 nanoplatelet aliquots over the length of the reaction: 15s, 45s, 90s, 150s, 400s, 300s, 500s (D) the model-based visualization of the CdSe.50/CdS.50 nanoplatelet

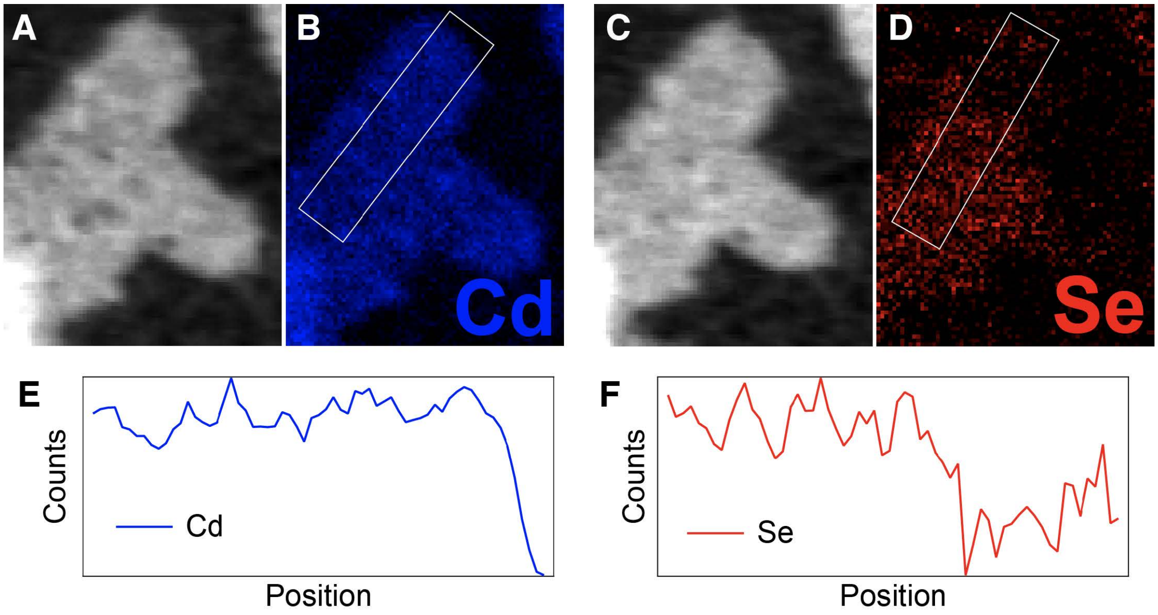

Figure S16. CdSe/CdS core/crown sample prepared from **15** and **8** A) Annular Dark Field (ADF) image, B) Cd M4,5 map, C) ADF image, D) Se L2/L3 map, E) cadmium map linescan, and F) selenium map linescan.

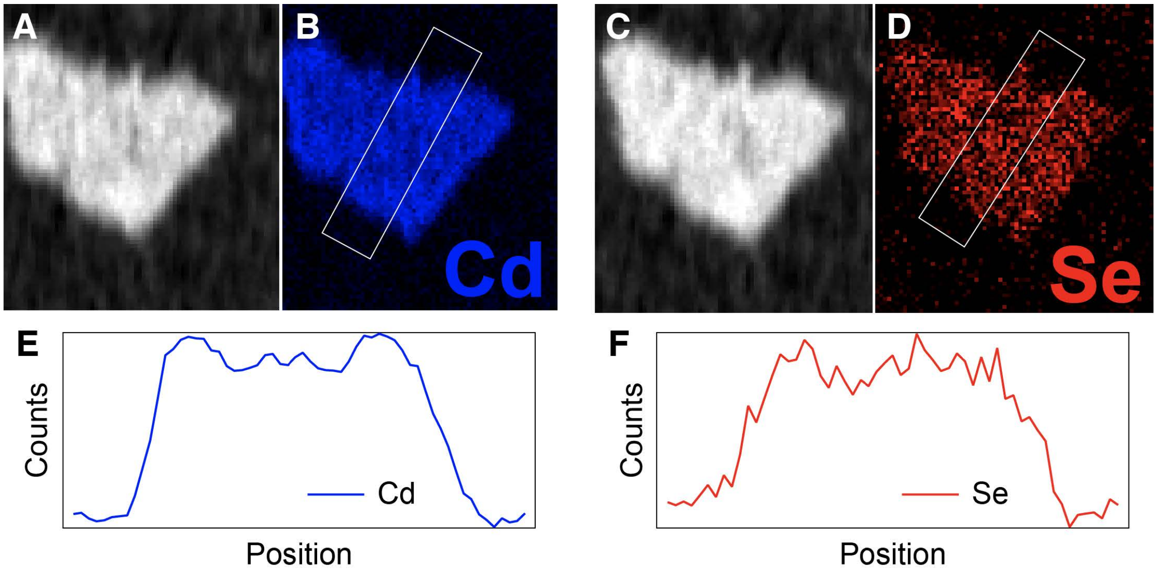

Figure S17. CdSe/CdS graded alloy prepared from **15** and **7** A) ADF image, B) Cd M4,5 map, C) ADF image, D) Se L2/L3 map, E) cadmium map linescan, and F) selenium map linescan.

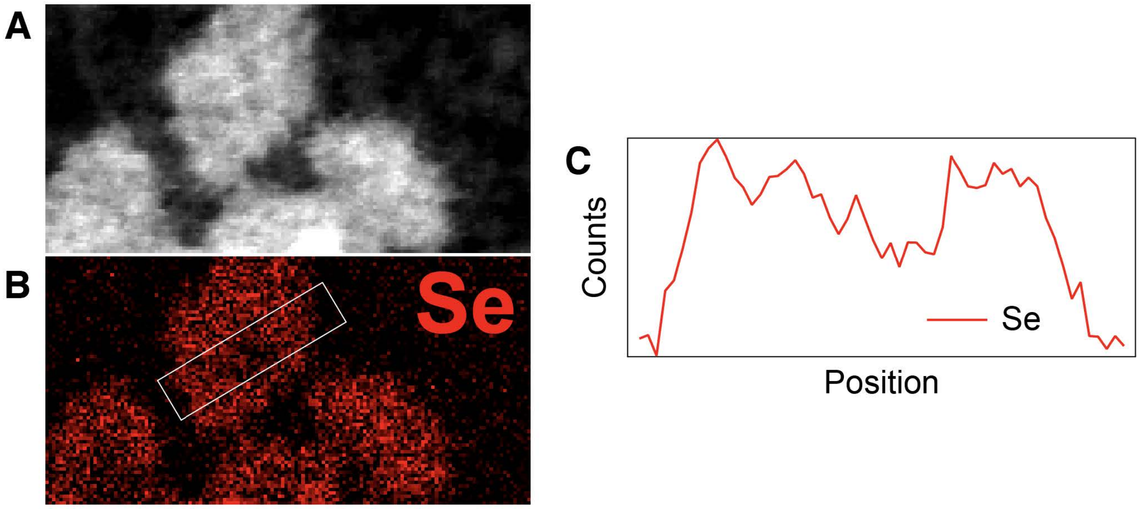

Figure S18. CdS/CdSe graded alloy sample prepared from **15** and **1** A) ADF image, B) Se L2/L3 map, C) selenium map linescan.

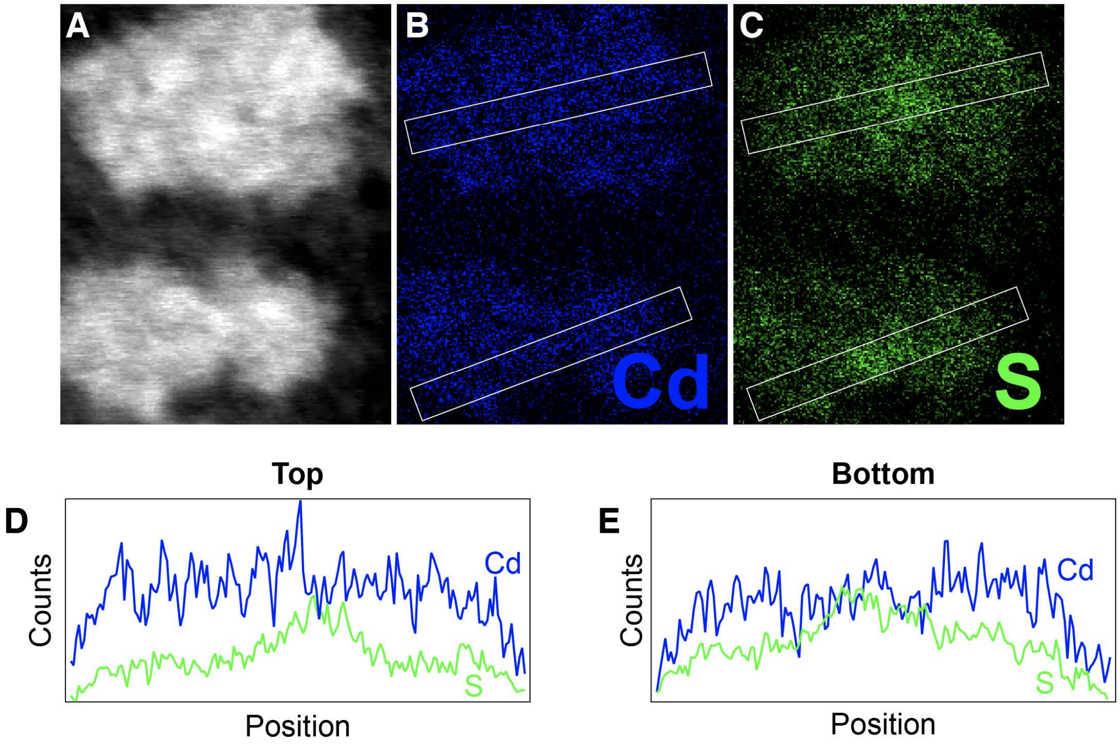

Figure S19. CdS/CdSe graded alloy sample prepared from **15** and **1** A) ADF image, B) Cd M4,5 map, C) S L2,3 map, D) cadmium and sulfur linescan (top) and E) cadmium and sulfur linescan (bottom).

References

1. Dufour, M.; Qu, J.; Greboval, C.; Méthivier, C.; Lhuillier, E.; Ithurria, S., Halide Ligands To Release Strain in Cadmium Chalcogenide Nanoplatelets and Achieve High Brightness. *ACS Nano* **2019,** *13* (5), 5326-5334.
2. Jana, S.; Frutos, M. d.; Davidson, P.; Abécassis, B., Ligand-induced twisting of nanoplatelets and their self-assembly into chiral ribbons. *Science Advances* **2017,** *3* (9), e1701483.
